# A synergistic multi-omics approach: causal sepsis drivers identified in activated CD4^+^ T cells by single-cell RNA sequencing and Mendelian randomization

**DOI:** 10.3389/fcimb.2026.1749207

**Published:** 2026-05-28

**Authors:** Jun Zhou, Yun Liu, Qiuyan Hu, Chengzhi Xu, Fei Yin, Ye Lu, Qiang Shi

**Affiliations:** Department of Emergency, Suzhou Ninth People’s Hospital, Suzhou Ninth Hospital Affiliated to Soochow University, Suzhou, China

**Keywords:** activated CD4+ T cells, CD52, Mendelian randomization, RPLP0, RPS15A, RPS18, sepsis, single-cell RNA sequencing

## Abstract

**Background:**

Sepsis is a life-threatening, heterogeneous syndrome with high mortality. This heterogeneity undermines current “one-size-fits-all” therapies and conventional biomarkers (e.g., PCT) lack prognostic power. A critical need exists to identify patient-specific endotypes and causal-driven therapeutic targets.

**Methods:**

We employed a synergistic multi-omics strategy to identify causal drivers of sepsis. First, we used single-cell RNA sequencing (scRNA-seq) on peripheral blood mononuclear cells (GSE175453) to identify the most pathologically relevant immune cell subpopulation. Next, we used marker genes from this subset as exposures in a two-sample Mendelian randomization (MR) study, using summary statistics from a large-scale sepsis GWAS (11,643 cases/474,841 controls; ieu-b-4980) and eQTL data (eQTLGen) to validate causal relationships. Findings were explored via in-silico functional-genomics (GSEA, GSVA) and validated via qPCR in a clinical cohort (5 sepsis vs. 5 healthy controls).

**Results:**

scRNA-seq analysis identified activated CD4^+^ T cells (Act.CD4T) as the cell subset contributing most significantly to sepsis pathogenesis. From 81 Act.CD4T marker genes, MR analysis identified four genes with a significant causal effect on sepsis risk: RPLP0 was identified as a causal risk factor (OR: 1.272; 95% CI: 1.031–1.569), while CD52 (OR: 0.903), RPS15A (OR: 0.954), and RPS18 (OR: 0.908) were identified as causal protective factors (all $p<0.05$). Clinical qPCR validation confirmed that RPLP0 was significantly upregulated in sepsis patients, while CD52, RPS15A, and RPS18 were downregulated. Functional analysis revealed these genes converge on a novel “Metabolism–Proteostasis–Immunity” regulatory axis, where RPLP0 drives a pathogenic program (HIF-1, IL-17, ERS) and RPS15A/RPS18 mediate a protective program (AMPK, mTORC1 inhibition).

**Conclusion:**

This study is to integrate scRNA-seq and MR to discover cell-specific causal genes for sepsis. The identified four-gene signature provides potentially robust, causally-validated biomarkers for patient stratification and reveals the “Metabolism–Proteostasis–Immunity” axis as a critical, therapeutically-targetable node in sepsis pathogenesis.

## 1 Introduction

Sepsis represents a formidable global health challenge, responsible for an estimated 48.9 million cases and 11 million deaths annually (Fleischmann-Struzek et al., 2020; Rudd et al., 2020), accounting for up to 50% of all in-hospital fatalities. Pathophysiologically, sepsis is not a monolithic disease but a heterogeneous syndrome driven by a dysregulated host response to infection (Singer et al., 2016; Van Der Poll et al., 2017). This complexity manifests as profound patient-to-patient variability in immune cell dynamics and molecular signatures (Davenport et al., 2016; Arora et al., 2023), which undermines the efficacy of current one-size-fits-all therapeutic strategies. Consequently, conventional biomarkers such as procalcitonin (PCT) and interleukin-6 (IL-6) exhibit limited specificity and prognostic power (Pierrakos and Vincent, 2010; Barichello et al., 2022), failing to capture the underlying cellular heterogeneity. This critical knowledge gap highlights an urgent need to deconstruct the intricate molecular and cellular architecture of sepsis to identify robust, causally-validated targets for precision medicine (Hasin et al., 2017).

The advent of high-throughput technologies offers unprecedented opportunities to address this challenge (Reyes et al., 2020). Single-cell RNA sequencing (scRNA-seq), in particular, enables the dissection of complex immune landscapes at unparalleled resolution, allowing for the identification of specific cell subpopulations and gene expression programs that drive disease pathogenesis (Zhang et al., 2022). However, while scRNA-seq can reveal a multitude of disease-associated genes, it cannot distinguish causal drivers from downstream bystanders or spurious correlations (Davies et al., 2018). To bridge this critical gap between association and causation, Mendelian randomization (MR) provides a powerful analytical framework. By leveraging randomly allocated genetic variants as instrumental variables, MR can formally test for a causal relationship between gene expression and disease risk, thereby mitigating the confounding and reverse causation biases that plague conventional observational studies (Zhu et al., 2016).

The synergistic integration of scRNA-seq for hypothesis generation and MR for causal validation thus offers a robust pipeline for discovering bona fide therapeutic targets (Yan et al., 2017). Despite the individual power of these approaches, their systematic integration to dissect the causal regulatory networks in sepsis remains a largely unexplored frontier (Giamarellos-Bourboulis et al., 2024). In this study, we employ this synergistic multi-omics strategy to identify the key immune cell types and causal genetic drivers of sepsis. Our analysis pinpoints activated CD4^+^ T cells as the most pathologically relevant immune subset. Within this population, we identify four novel genes with significant causal effects on sepsis risk: RPLP0 as a risk factor, and CD52, RPS15A, and RPS18 as protective factors. Functional interrogation of these genes reveals their convergence on critical biological pathways, leading us to propose a novel “Metabolism–Proteostasis–Immunity” regulatory axis in sepsis pathogenesis. These findings provide not only new molecular biomarkers for patient stratification but also promising, causally-supported targets for future therapeutic intervention.

## 2 Materials and methods

### 2.1 Data acquisition

**Figure 1:**
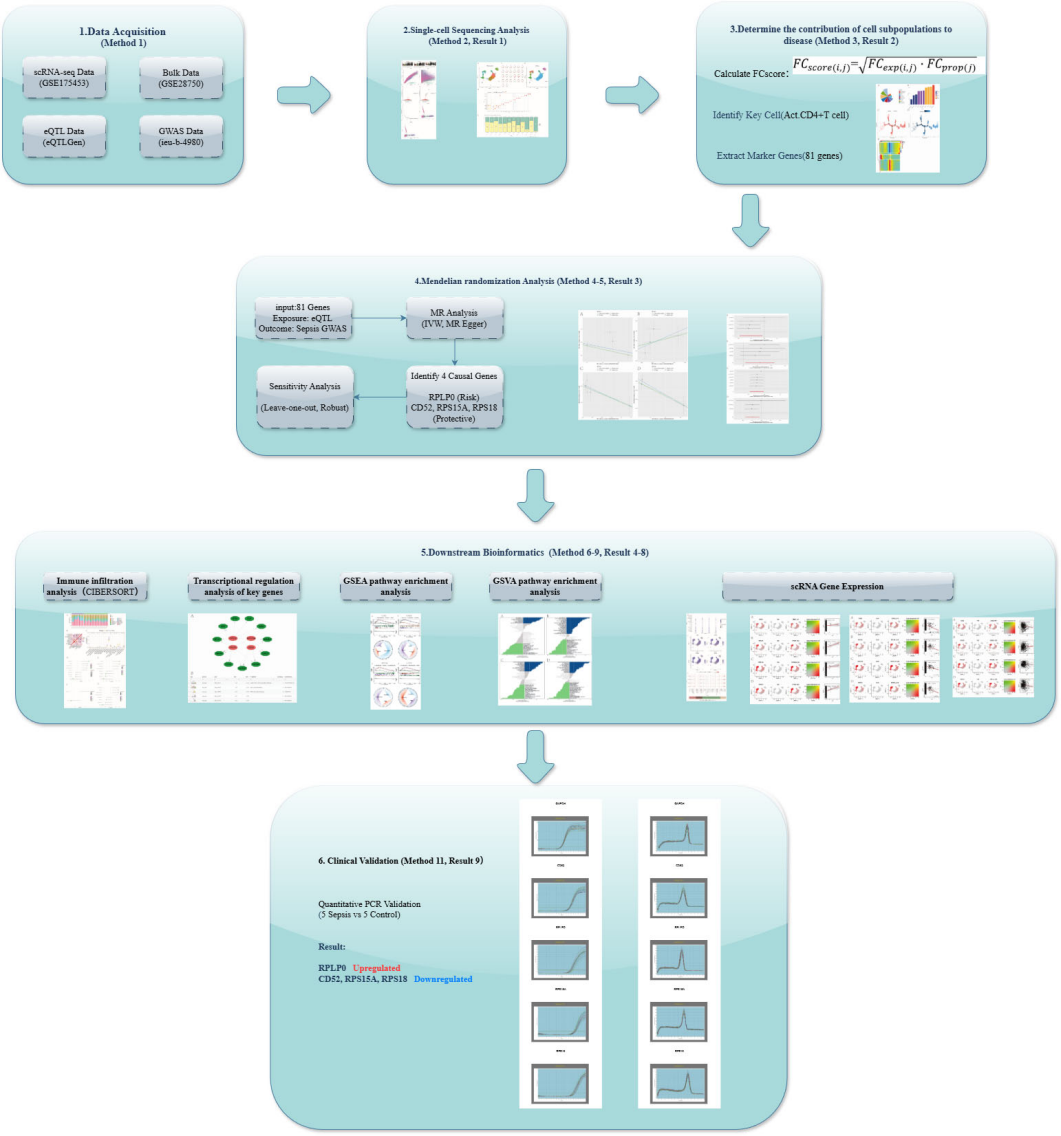
Synergistic multi-omics workflow for identifying causal genetic drivers of sepsis.

1. The Gene Expression Omnibus (GEO) is a public gene expression database maintained by the National Center for Biotechnology Information (NCBI) (https://www.ncbi.nlm.nih.gov/geo/info/datasets.html). We downloaded the single-cell data file for GSE175453 from the GEO database. Sample data from 9 cases with complete single-cell expression profiles were selected for analysis, comprising 5 control cases and 4 disease cases. Additionally, the Series Matrix File for GSE28750 was downloaded from GEO, which used the GPL570 annotation platform. This dataset included expression profile data from 30 patients, consisting of 20 control cases and 10 disease cases.
2. Exposed data: eQTL data comes from the eQTLGen consortium (https://www.eqtlgen.org) database. The eQTLGen consortium aims to study the genetic structure of blood gene expression and understand the genetic basis of complex traits. The large-scale eQTLGen project is currently in its second phase and is focused on conducting large-scale genome-wide meta-analyses in blood.
3. Outcome data: Participants in the outcome-related GWAS studies selected in this study were mainly people of European ancestry. Outcome summary data are all derived from the ieu database (ieu-b-4980). To date, the GWAS Catalog contains publications, top associations, and complete summary statistics. GWAS Catalog data are currently mapped to Genome Assembly and dbSNP Build. Of these, there were 11,643 cases and 474,841 controls in sepsis (**Figure 1**).

### 2.2 Single cell sequencing analysis

First, the expression profile was read via the Seurat package and filter out abnormal expression samples based on the UMI count, gene number and mitochondrial gene ratio captured in each cell; then data were standardized, normalized, and subjected to PCA linear dimensionality reduction, and then use Scree plot to observe the optimal number of PCs, and obtain the positional relationship between each cluster through UMAP nonlinear dimensionality reduction; use CellMarker and PanglaoDB databases and query the literature to find the cell types existing in the corresponding tissue and the corresponding marker genes for cell annotation.

### 2.3 Determine the contribution of cell subpopulations to disease

We characterize the contribution of different cell subpopulations to disease by considering changes in cell number and gene expression. First, we identified characteristic genes for each subpopulation. Briefly, we performed bulk differential gene expression analysis and characterized the changes in this process by identifying differential genes from the disease group to the control group. Then, we defined FCscore, which measures changes in the number and expression levels of characteristic genes in biological processes.


$$FC_{score(i,j)}=\sqrt{FC_{\exp(i,j)}\cdot FC_{prop(j)}}$$

Among them, FCexp(i,j) represents the expression fold change of the i-th characteristic gene in the j-th cluster. FCprop(j) represents the proportional fold change of the j-th cell cluster, calculated as the ratio of the cluster’s percentage in the sepsis group relative to the control group. The FCscore(i,j) is defined as the square root of the product of these two variables to reflect the integrated contribution of both gene expression and cell abundance shifts to the disease state.

### 2.4 Mendelian randomization analysis

Select the SNPs associated with the locus-wide significance threshold (P < 1e-5) of each gene as potential IVs (Instrumental variables) (Birney, 2021), calculate the LD (linkage disequilibrium) between SNPs, and calculate the LD (linkage disequilibrium) between SNPs at R2 < 0.001 (clumping Among the SNPs with window size=10,000kb), only SNPs with p2 < 5e-5 are retained (Larsson et al., 2023). In turn, it passes through Inverse variance weighted (IVW, using meta-analysis method combined with the Wald estimate of each SNP), MR Egger (based on the assumption that the instrument strength is independent of direct effects (InSIDE)), Weighted median (the weighted median method allows up to Correctly estimate causality in 50% of cases where IVs are invalid), Weighted mode (weighted model estimation has greater ability to detect causal effects, smaller bias, and lower Type I error rate than MR-Egger regression) (Ding et al., 2023; Zhang et al., 2024). A statistical method (if there is only one statistical method for the SNP in the causal relationship, only the Wald ratio is used) to evaluate the reliability of the causal relationship to obtain an overall estimate of the impact of all cis and some cross-region gene expression on sepsis in whole blood. Finally, the screened causal relationships were verified and analyzed through methods such as heterogeneity (Cochran’s IVW Q test), genetic diversity test, and leave-one-out method (Xiao et al., 2022). Specifically, while Inverse Variance Weighted (IVW) provides the primary causal estimate, other methods including MR-Egger, Weighted Median, and Weighted Mode serve as robust sensitivity analyses to detect and adjust for potential pleiotropy. This multi-method approach ensures that the identified causal relationships are not biased by invalid genetic instruments or alternative biological pathways.

### 2.5 Sensitivity analysis

We used Mendelian Randomization (MR) leave-one-out sensitivity analysis to evaluate the impact of specific genetic variants on sepsis risk. This method identifies and eliminates variants that disproportionately affect the overall estimate by systematically excluding each SNP and recalculating the pooled effect size of the remaining SNPs. The removal of each SNP produces a new point estimate and its 95% confidence interval to assess the SNP’s unique contribution and robustness to the overall results. This chart summarizes the estimates after individual SNPs are removed, as well as the overall estimate when all SNPs are included. By comparing these estimates, we can observe the impact of removing any single SNP on the overall results to determine the robustness of our analysis (Carter et al., 2021).

### 2.6 Immune cell infiltration analysis

The CIBERSORT method is a widely used method to evaluate immune cell types in the microenvironment. This method is based on the principle of support vector regression and performs deconvolution analysis on the expression matrix of immune cell subtypes (Craven et al., 2021). It contains 547 biomarkers that distinguish 22 human immune cell phenotypes, including T cells, B cells, plasma cells, and myeloid cell subsets. This study uses the CIBERSORT algorithm to analyze patient data to infer the relative proportions of 22 types of immune infiltrating cells, and perform spearman correlation analysis on gene expression and immune cell content.

### 2.7 Transcriptional regulation analysis of key genes

This study used the R package “RcisTarget” to predict transcription factors (He et al., 2023). All calculations performed by RcisTarget are based on motifs. The normalized enrichment score (NES) of a motif depends on the total number of motifs in the database. TFs in RcisTarget are inferred through genome-wide motif enrichment analysis (AUC and NES scores) based on comprehensive public databases such as cisBP and JASPAR, enabling systematic regulatory network identification independent of specific experimental conditions. While this approach provides broader coverage than individual experimental datasets, it is primarily predictive, and we have utilized it here as high-confidence supplementary evidence for mechanistic exploration. In addition to the motifs annotated by the source data, we inferred further annotation files based on motif similarity and gene sequence. The first step in estimating the overexpression of each motif on a gene set is to calculate the area under the curve (AUC) for each motif-motif set pair. This analysis was conducted using recovery curve calculations derived from the gene set relative to the motif ordering. The normalized enrichment score (NES) for each motif was computed based on the area under the curve (AUC) distribution across all motifs in the gene set (Yan, 2025).

### 2.8 GSEA pathway enrichment analysis

GSEA was used to further analyze the differences in signaling pathways between high and low expression groups. The background gene set is the version 7.0 annotated gene set downloaded from the MsigDB database. As an annotated gene set for subtype pathways, differential expression analysis of pathways between subtypes is performed, and significantly enriched gene sets (adjusted p<0.05) to sort. GSEA analysis is often used in studies that closely combine disease classification and biological significance (Ding et al., 2024).

### 2.9 Gene set difference analysis

Gene set variation analysis (GSVA) ​​is a non-parametric, unsupervised method for assessing transcriptome gene set enrichment. GSVA converts gene-level changes into pathway-level changes by comprehensively scoring the gene set of interest, and then determines the biological function of the sample (Cao et al., 2024). This study will download gene sets from the Molecular signatures database, and use the GSVA algorithm to comprehensively score each gene set to evaluate potential biological function changes in different samples.

### 2.10 Statistical analysis

Reliable MR analysis is based on three premise assumptions: (1) correlation assumption (the instrumental variable is closely related to the exposure, but not directly related to the outcome), (2) independence assumption (the instrumental variable cannot be related to the confounding factors), (3) Exclusivity hypothesis (instrumental variables can only affect outcomes through exposure (Dudbridge, 2021). When IV can affect outcomes through other pathways, it is determined that gene pleiotropy exists). In this analysis, R language (version 4.2.2) was used. All statistical tests were two-sided, and p<0.05 was considered statistically significant.

### 2.11 Quantitative PCR validation

RNA extraction & quality control: Total RNA was isolated from peripheral blood samples (5 sepsis patients vs. 5 healthy controls, summary of the clinical characteristics for these patients is in the **Supplementary Table 1**) using the Total RNA Extraction Kit (Nuolan Biotechnology, China). High-purity RNA (RIN ≥7.0, Agilent 2100 Bioanalyzer) was obtained via tissue homogenization, chloroform phase separation, ethanol precipitation, and silica-membrane purification. All samples exhibited A260/A280 ratios of 1.87–2.28 and concentrations of 433.7–532.2 ng/μL.

Reverse transcription & qPCR: cDNA synthesis employed the Universal Reverse Transcription Kit with 1 μg total RNA under thermocycling: 30 °C (30 min) → 42 °C (30 min) → 85 °C (10 min). Target genes (CD52, RPLP0, RPS15A, RPS18) and reference gene (GAPDH) were amplified using ComSYBR qPCR Mix on an Agilent MX3000P system. Reaction mix (20 μL): 0.08 μM primers, 2 μL cDNA, Taq DNA polymerase. Cycling: 95 °C (3 min); 40 cycles of 95 °C (12 s) → 62 °C (40 s). Primer sequences are listed in the **Supplementary Table 2**. Specificity confirmed by BLAST; amplification efficiency R²>0.99. Data analysis: Relative gene expression was calculated via 2^(-ΔΔCt) method using GAPDH for normalization and healthy controls for calibration. Inter-group differences were assessed by two-tailed unpaired Student’s t-test (P<0.05) (Ma et al., 2021).

## 3 Results

### 3.1 Single cell level analysis in scRNA-Seq data

**Figure 2:**
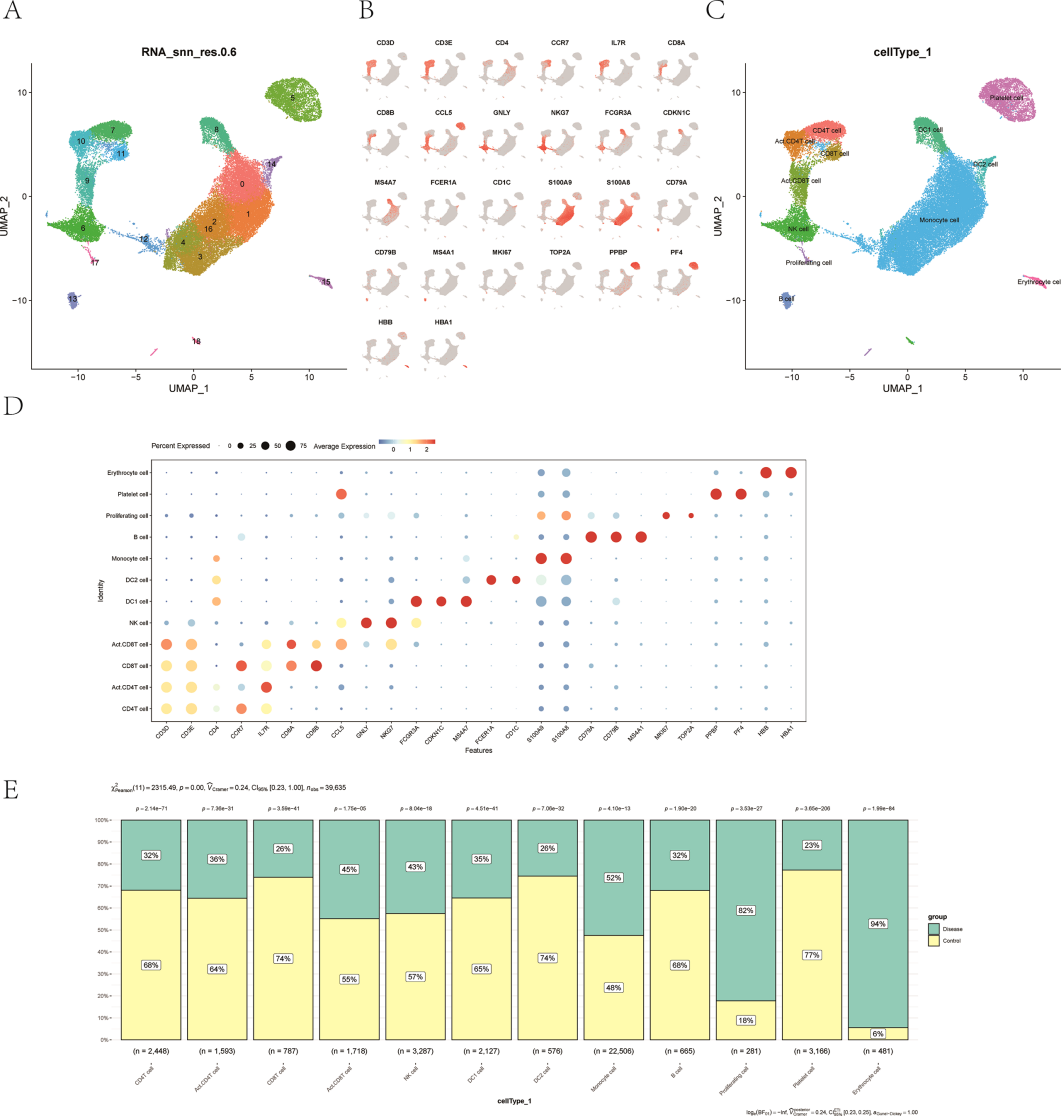
Cell annotation and classification profile. **(A)** UMAP visualization of cell clusters. Cells were grouped into 19 distinct clusters using the UMAP algorithm based on the significant principal components (PCs) identified from PCA. **(B, C)** Cell annotation of the 19 clusters. The clusters were annotated into 12 major cell types: CD4+ T cells, Activated CD4+ T (Act.CD4T) cells, CD8+ T cells, Activated CD8+ T (Act.CD8T) cells, NK cells, DC1 cells, DC2 cells, Monocytes, B cells, Proliferating cells, Platelets, and Erythrocytes. **(D)** Dot plot illustrating the expression of specific signature markers across the 12 cell types. The size of each dot represents the percentage of cells expressing the marker, while the color intensity indicates the average expression level. **(E)** Comparison of the relative proportions of the 12 cell types between the two study groups.

Expression profiles were first read in via the Seurat package, where we filtered cells based on the total number of UMIs per cell, the number of genes expressed, and the percentage of mitochondrial reads per cell. Where outliers are defined as three median absolute deviations (MAD) from the median. And filter out low-expressing cells based on violin plots and scatter plots (nFeature_RNA > 200 & percent.mt < 3*MAD & nFeature_RNA < 3*MAD & nCount_RNA < 3*MAD), and then use the DoubletFinder package to filter double cells, retaining a total of 39,635 cells, filtered violin plots, scatter plots (**Supplementary Figures 1A, B**). The data were subjected to standardization, homogenization, PCA, and harmony analysis in sequence (**Supplementary Figures 1C–F**). The positional relationship between each cluster was obtained through UMAP analysis, and there were a total of 19 cell clusters (**Figure 2A**). This study further annotated these 19 cell clusters (**Figure 2B**), which were annotated to CD4T cell, Act.CD4T cell, CD8T cell, Act.CD8T cell, NK cell, DC1 cell, DC2 cell, Monocyte cell, and B cell., Proliferating cell, Platelet cell, Erythrocyte cell, these 12 cell categories (**Figure 2C**). Bubble chart of classic markers of 12 cells (**Figure 2D**) and histogram of cell proportions corresponding to groups (**Figure 2E**).

### 3.2 Determine the contribution of cell subpopulations to disease

**Figure 3:**
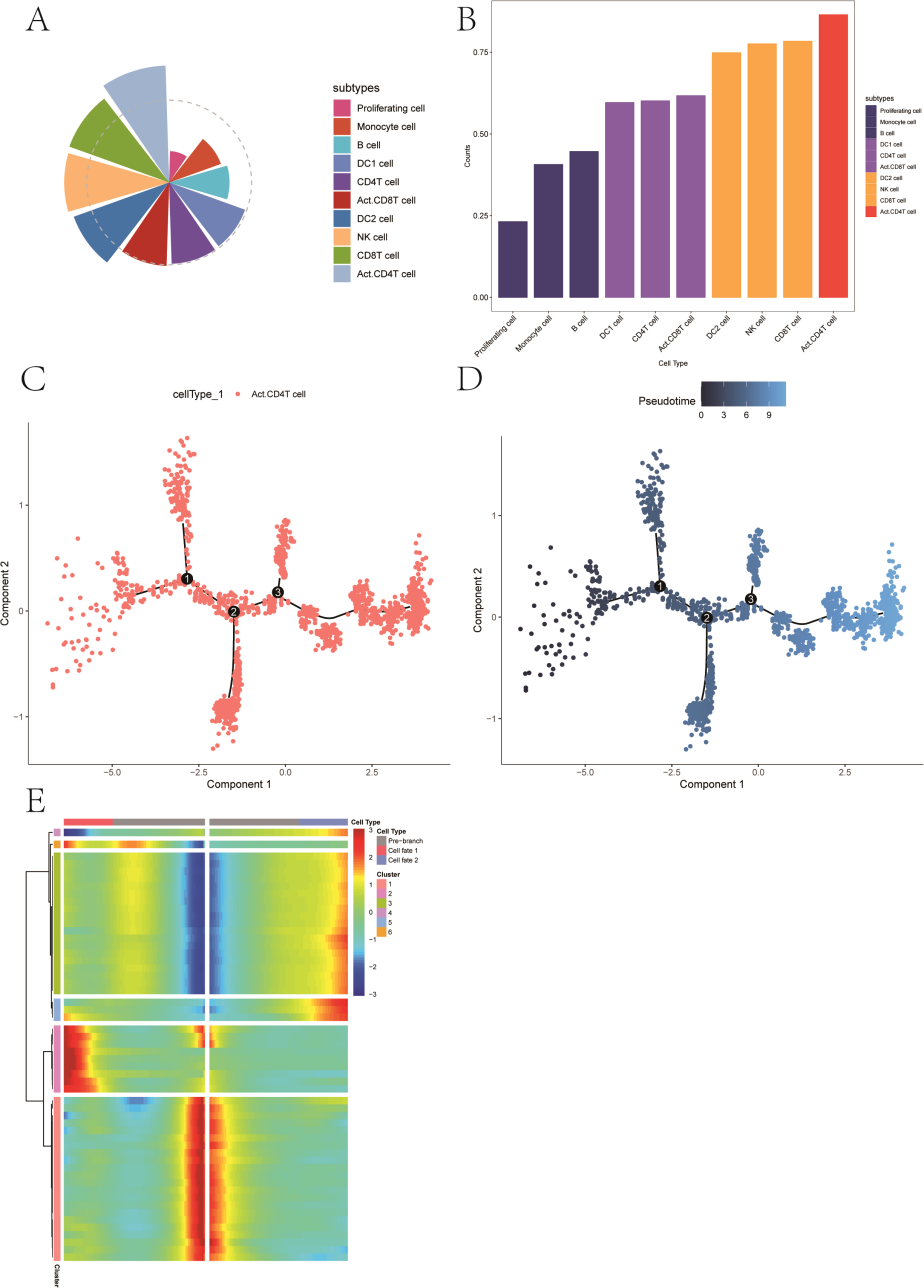
Cell subpopulation contribution and developmental trajectories. **(A)** Proportional distribution across different cell subsets. **(B)** Comparison of the total number of interactions in the cell-cell communication networks among the 12 cell types. The interaction frequency increases from left to right, with Activated CD4+ T (Act.CD4T) cells exhibiting the highest total interaction strength. **(C, D)** Pseudotime analysis and reconstruction of cell developmental trajectories. **(E)** Gene expression dynamics along different cell trajectory branches.

We removed Platelet cells and Erythrocyte cells (considering that red blood cells and platelets do not have nuclei), and used the remaining cells to characterize the contribution of different cell subpopulations to sepsis by considering changes in cell number and gene expression. First, we selected the top 12 (logFC > 1) differential genes from the disease group to the control group to describe the changes in this process. Then, we defined FCscore, which measures changes in the number and expression levels of characteristic genes in biological processes. Finally, it was found that Act.CD4T cells contribute the most to sepsis (**Figures 3A, B**). At the same time, we extracted marker genes (CellMarkers.txt) unique to each cell subtype from the single-cell data through the FindAllMarkers function. The filtering conditions were avg_log2FC>1 and p_val_adj<0.05. Act.CD4T cell has a total of 81 genes. These 81 marker genes are the preliminary genes for our next step of Mendelian randomization. In addition, we extracted Act.CD4T cells and showed the cell trajectory analysis results of Act.CD4T cells (**Figures 3C–E**). Cell trajectory analysis was employed to reconstruct the dynamic transition of CD4^+^ T cells from a steady state to a pathologically activated phenotype, providing a temporal dimension to the immune dysregulation observed in sepsis.

### 3.3 Mendelian randomization analysis

**Figure 4:**
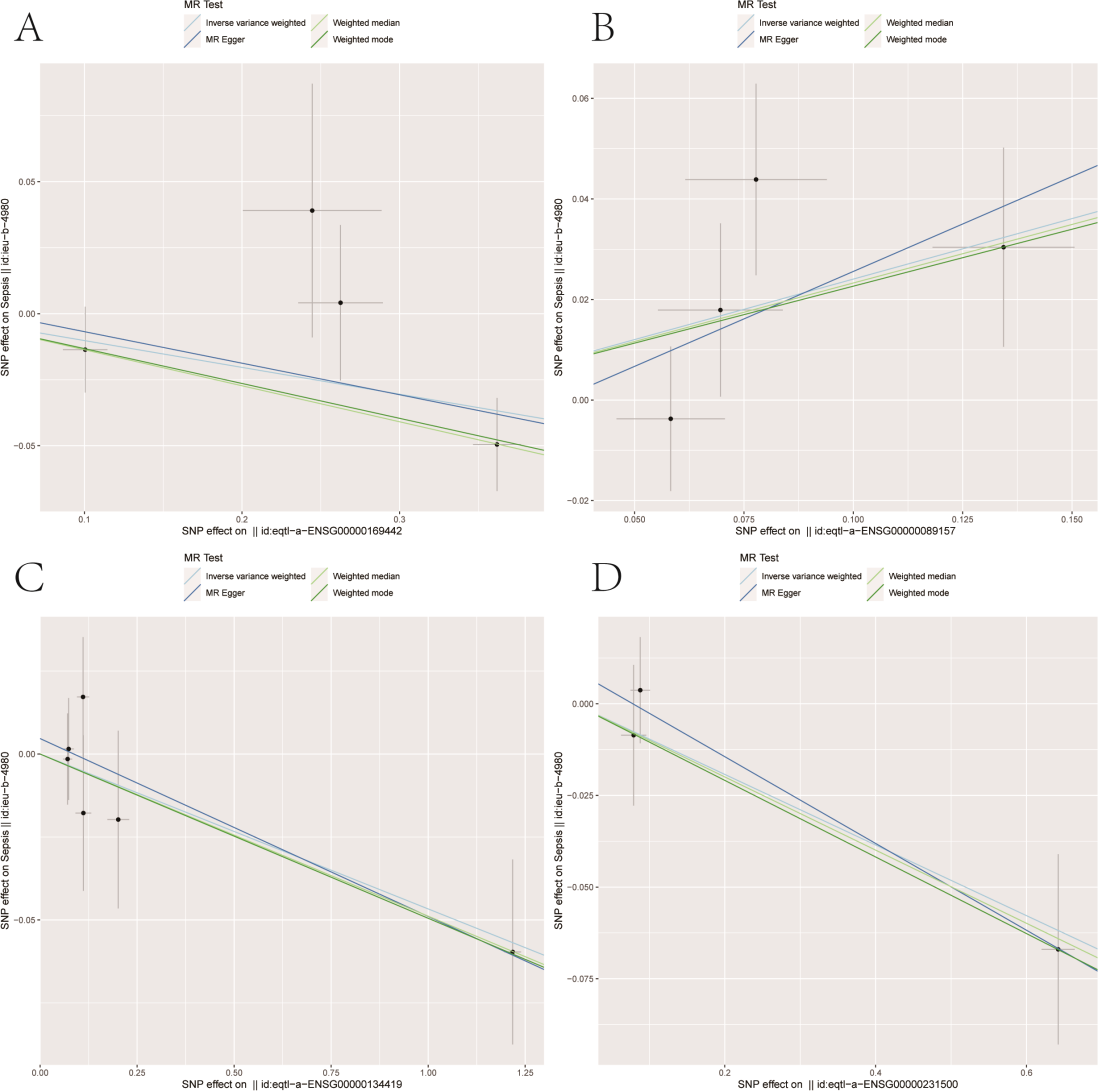
Mendelian randomization (MR) analysis. **(A–D)** Causal estimates for the four gene-sepsis pairs. The plots display the causal effect sizes, expressed as Odds Ratios (ORs) with 95% confidence intervals, derived from Mendelian Randomization analysis for the identified risk and protective genes.

**Figure 5:**
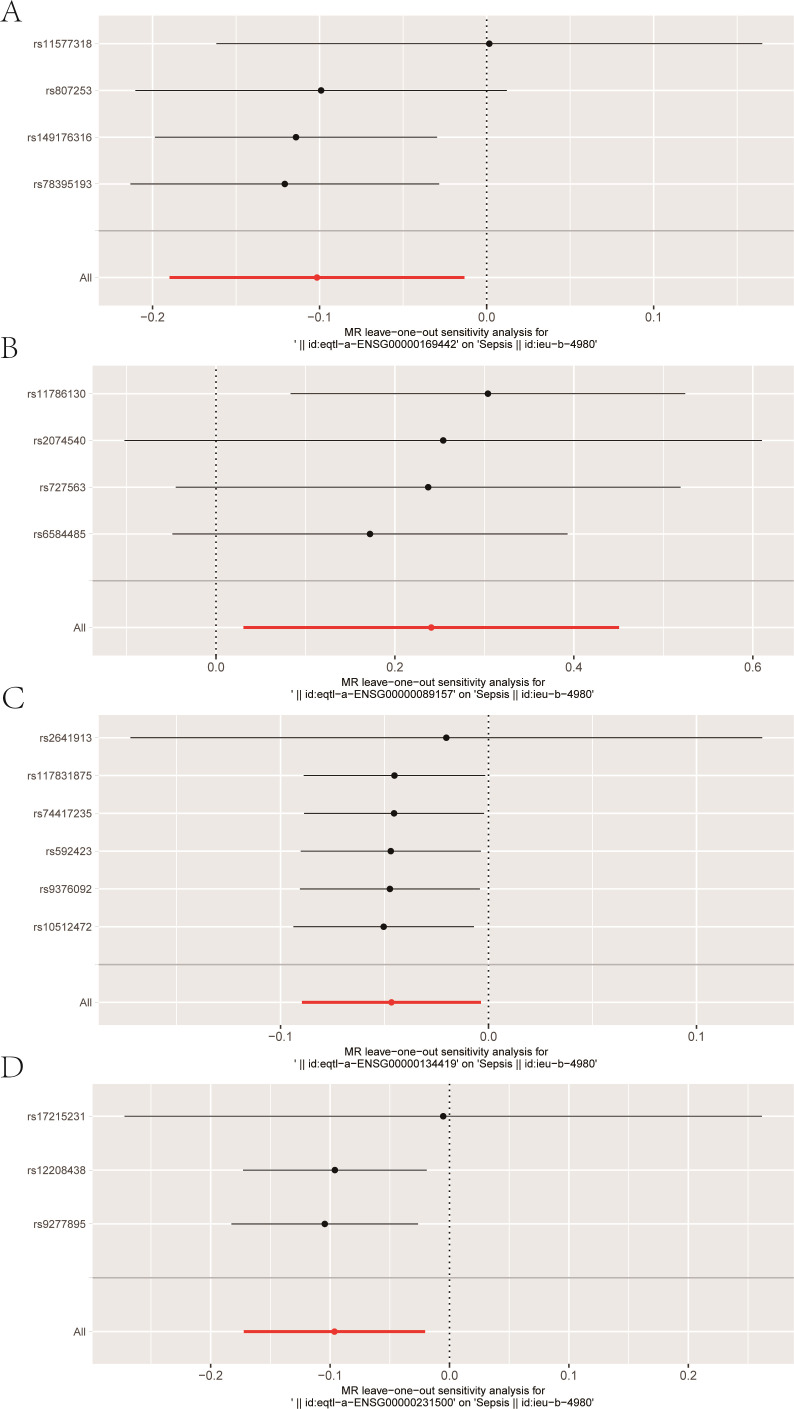
Leave-one-out sensitivity analysis. **(A–D)** Forest plots of the leave-one-out analysis for SNPs associated with the candidate genes. Each plot illustrates the stability of the causal estimate by systematically excluding one SNP at a time to ensure that the overall effect is not driven by a single influential outlier.

In order to find out the key genes that affect sepsis, we used the 81 marker genes obtained in the previous step and obtained the outcome id through the summary statistics of 486,484 sepsis-related samples (Controls: 474,841; Cases: 11,643): ieu - b - 4980. Use extract_instruments and extract_outcome_data in sequence to extract 72 pairs of genes with causal relationships related to outcomes (**Supplementary Table 3**), and screen out 4 pairs of genes with causal relationships corresponding to eQTL positive outcomes (**Figure 4**) (IVW pval < 0.05). The corresponding genes are: CD52, RPLP0, RPS15A, and RPS18. Among them, RPLP0 (1.272; 1.031 −1.569; p=0.024) may be associated with a high risk of sepsis. And CD52 (0.903; 0.827−0.987; p=0.024), RPS15A (0.954; 0.914−0.996; p=0.033), RPS18 (0.908; 0.842-0.980; p=0.012) may be associated with a lower risk of sepsis. The causal relationships of the four genes were further analyzed through sensitivity analysis using the leave-one-out method to determine their reliability. The results showed that excluding any SNP had no obvious impact on the overall error bar. This shows that the four pairs of causal relationships we selected are robust (**Figure 5**). Therefore, these four genes are the key genes for our subsequent research.

### 3.4 Immune infiltration analysis

**Figure 6:**
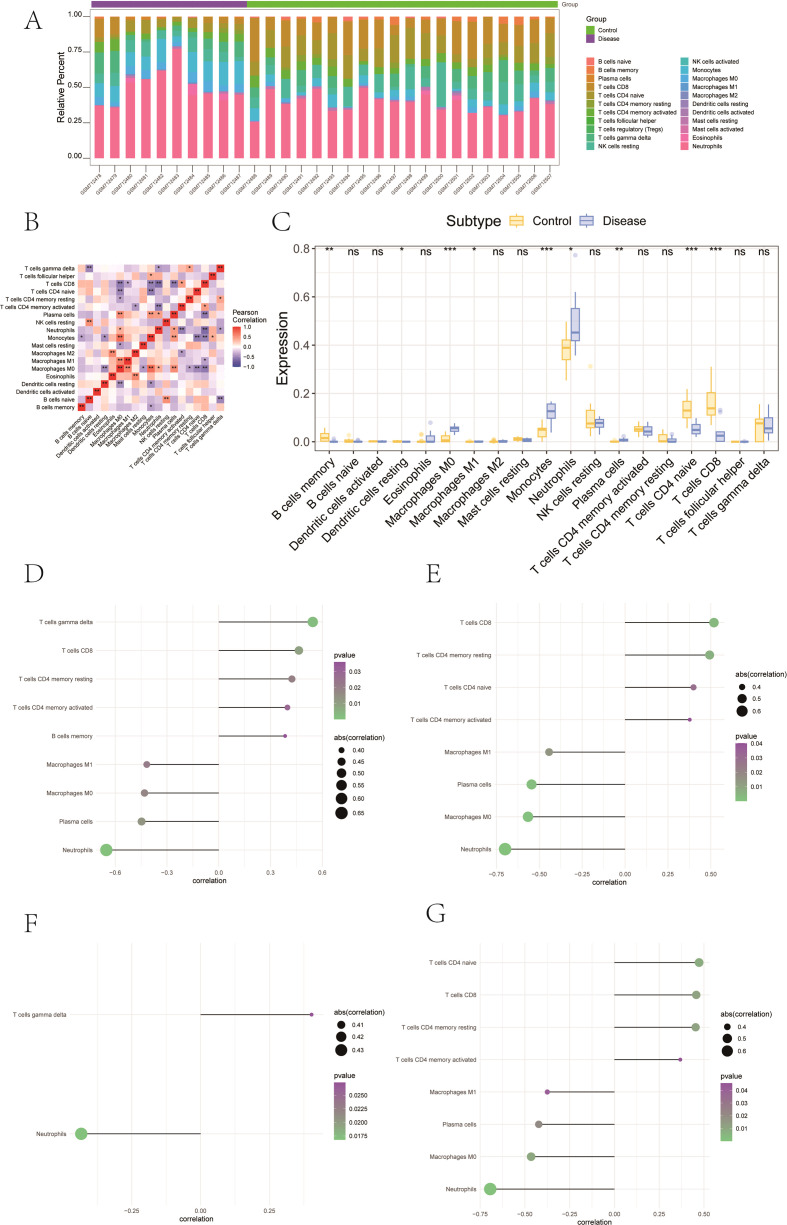
Immune infiltration analysis. **(A)** Relative proportions of immune cell subpopulations in the control and sepsis groups. **(B)** Correlation matrix illustrating the associations between different immune cell types. Red indicates a positive correlation, and blue indicates a negative correlation; the intensity of the color and the size of the circles correspond to the strength of the correlation coefficient. **(C)** Comparison of immune cell abundance between the control and sepsis groups. Blue represents the control group, while yellow represents the sepsis group. (Note: These results are based on CIBERSORT deconvolution of bulk transcriptomic data). **(D–G)** Correlation analysis between the identified candidate genes and the infiltration levels of various immune cell types. *: p<0.05, **: P<0.01, ***: P<0.001, ns: not significant.

The microenvironment is mainly composed of immune cells, extracellular matrix, various growth factors, inflammatory factors and special physical and chemical characteristics, which significantly affects the diagnosis of diseases and the sensitivity of clinical treatment. We further explored the potential molecular mechanisms by which key genes affect the progression of sepsis by analyzing the relationship between key genes and immune infiltration in the sepsis data set. We demonstrated the proportion of immune cell content in each patient and the relationship between immune cells correlation (**Figures 6A, B**). In addition, the research results showed that there were significant differences in differential expression between groups in 9 cells including B cells memory, Dendritic cells resting, Macrophages M0, and Monocytes (**Figure 6C**). We further explored the relationship between key genes and immune cells and found that CD52 has a significant positive correlation with T cells gamma delta, T cells CD8, T cells CD4 memory resting, etc., and a significant negative correlation with Neutrophils, Plasma cells, Macrophages M0, etc. (**Figure 6D**); RPLP0 has a significant positive correlation with T cells CD8, T cells CD4 memory resting, T cells CD4 naive, etc., and a significant negative correlation with Neutrophils, Macrophages M0, Plasma cells, etc. (**Figure 6E**); RPS15A and T cells gamma delta has a significant positive correlation, and a significant negative correlation with Neutrophils (**Figure 6F**); RPS18 has a significant positive correlation with T cells CD4 naive, T cells CD8, T cells CD4 memory resting, etc., and has a significant positive correlation with Neutrophils, Macrophages M0, Plasma cells, etc. Significant negative correlation (**Figure 6G**). These correlations with diverse immune subsets indicate that while the causal significance of these genes is anchored in CD4^+^ T cells, they likely participate in systemic immune crosstalk, reflecting the broader dysregulated microenvironment in sepsis.

### 3.5 Transcriptional regulation analysis of key genes

**Figure 7:**
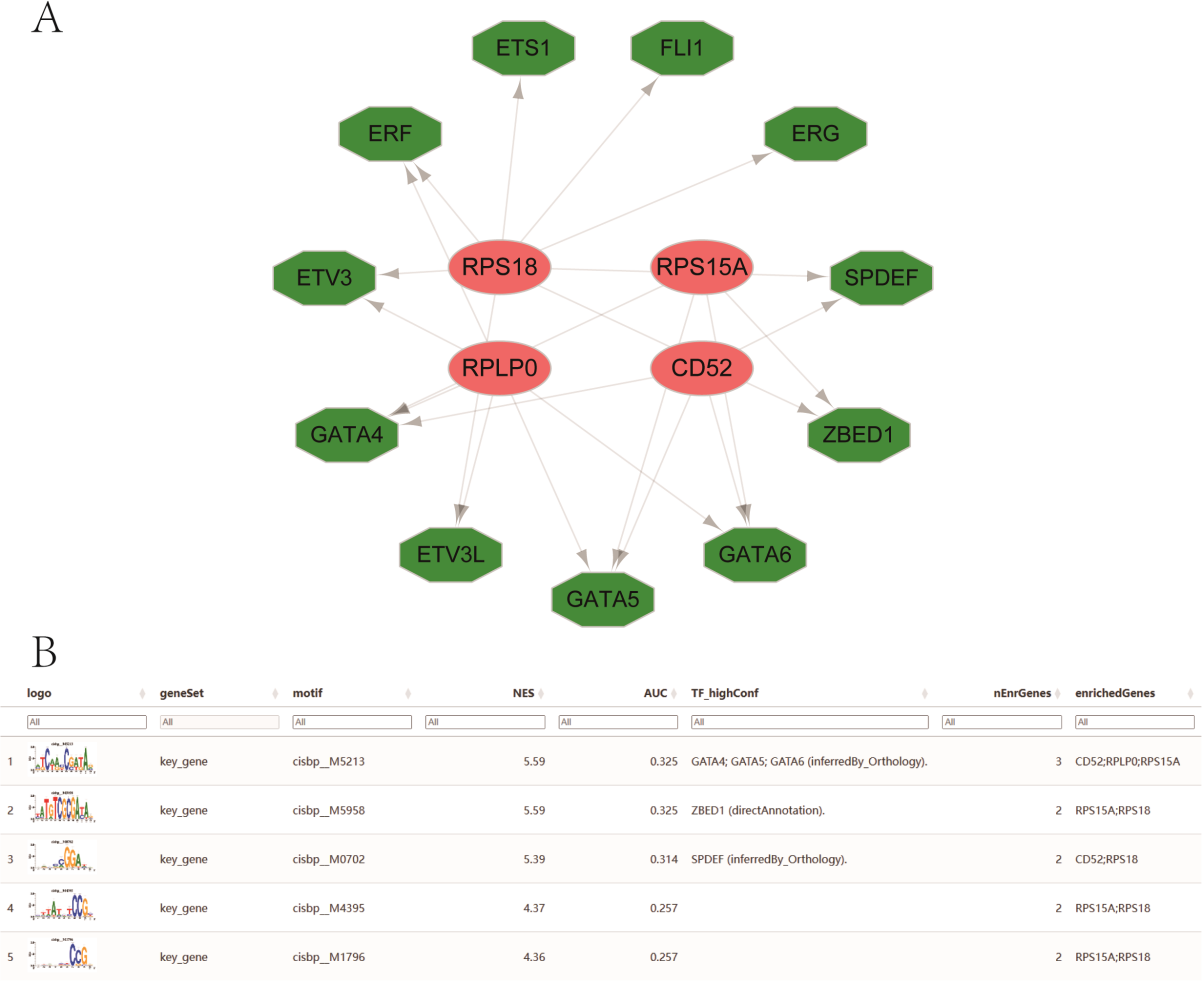
Transcriptional regulatory network of key genes. **(A)** Regulatory network between key genes and transcription factors (TFs). Red nodes represent the key genes, and green nodes represent the predicted transcription factors. **(B)** Visualization of enriched motifs and their corresponding transcription factors associated with the key genes. (This analysis was performed using the RcisTarget algorithm).

We used these four key genes as the gene set for this analysis and found that they are regulated by common mechanisms such as multiple transcription factors. Therefore, enrichment analysis of these transcription factors was performed using cumulative recovery curves. Motif-TF annotation and selection analysis of important genes showed that the Motif with the highest normalized enrichment score (NES: 5.59) is cisbp:M5213. We display all enriched motifs and corresponding transcription factors of key genes (**Figure 7**).

### 3.6 GSEA pathway enrichment analysis

**Figure 8:**
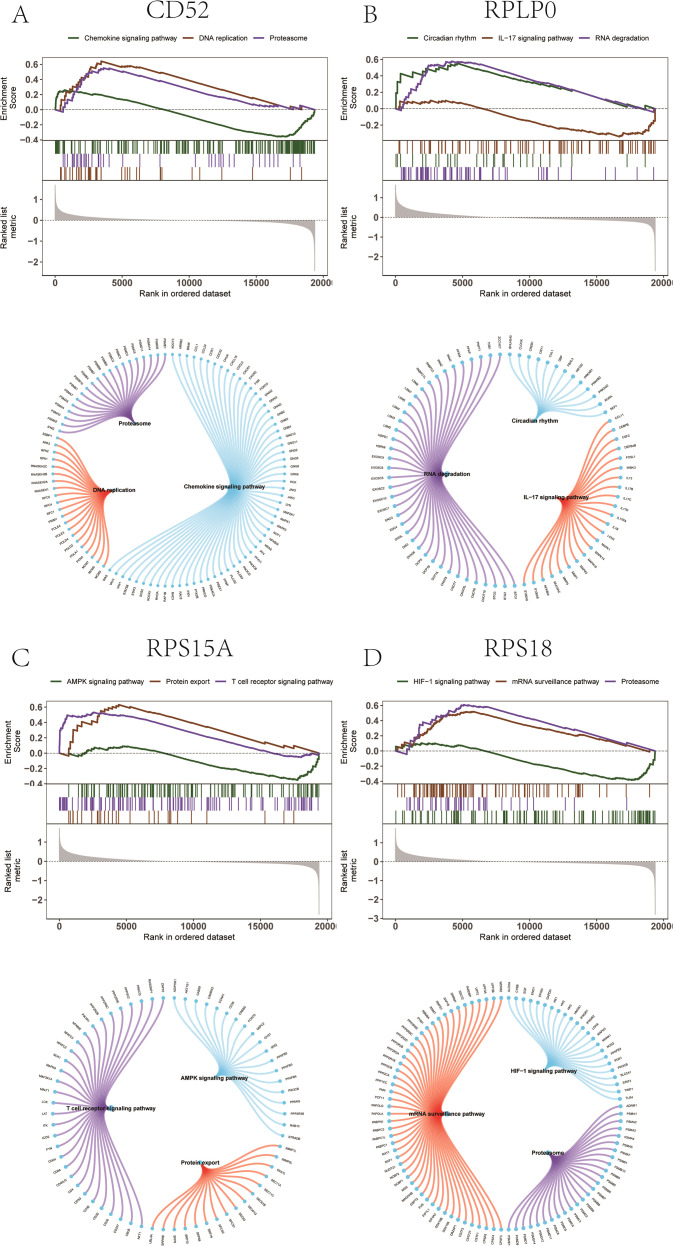
Gene Set Enrichment Analysis (GSEA) of candidate genes. **(A–D)** KEGG signaling pathways significantly enriched for the identified candidate genes. The plots illustrate the specific signaling pathways involved, their regulatory directions (up-regulation or down-regulation), and the constituent genes contributing to the enrichment.

Next, we will study the specific signaling pathways enriched in 4 key genes to explore the potential molecular mechanisms by which key genes affect the progression of sepsis. The GSEA results show that the pathways enriched by CD52 included the chemokine signaling pathway, DNA replication, Proteasome and other pathways (**Figure 8A**); The pathways enriched by RPLP0 include Circadian rhythm, IL-17 signaling pathway, RNA degradation and other pathways (**Figure 8B**); The pathways enriched by RPS15A include AMPK signaling pathway, Protein export, T cell receptor signaling pathway and other pathways (**Figure 8C**); the pathways enriched by RPLP0 include HIF-1 signaling pathway, mRNA surveillance pathway, Proteasome and other pathways (**Figure 8D**).

### 3.7 GSVA pathway enrichment analysis

**Figure 9:**
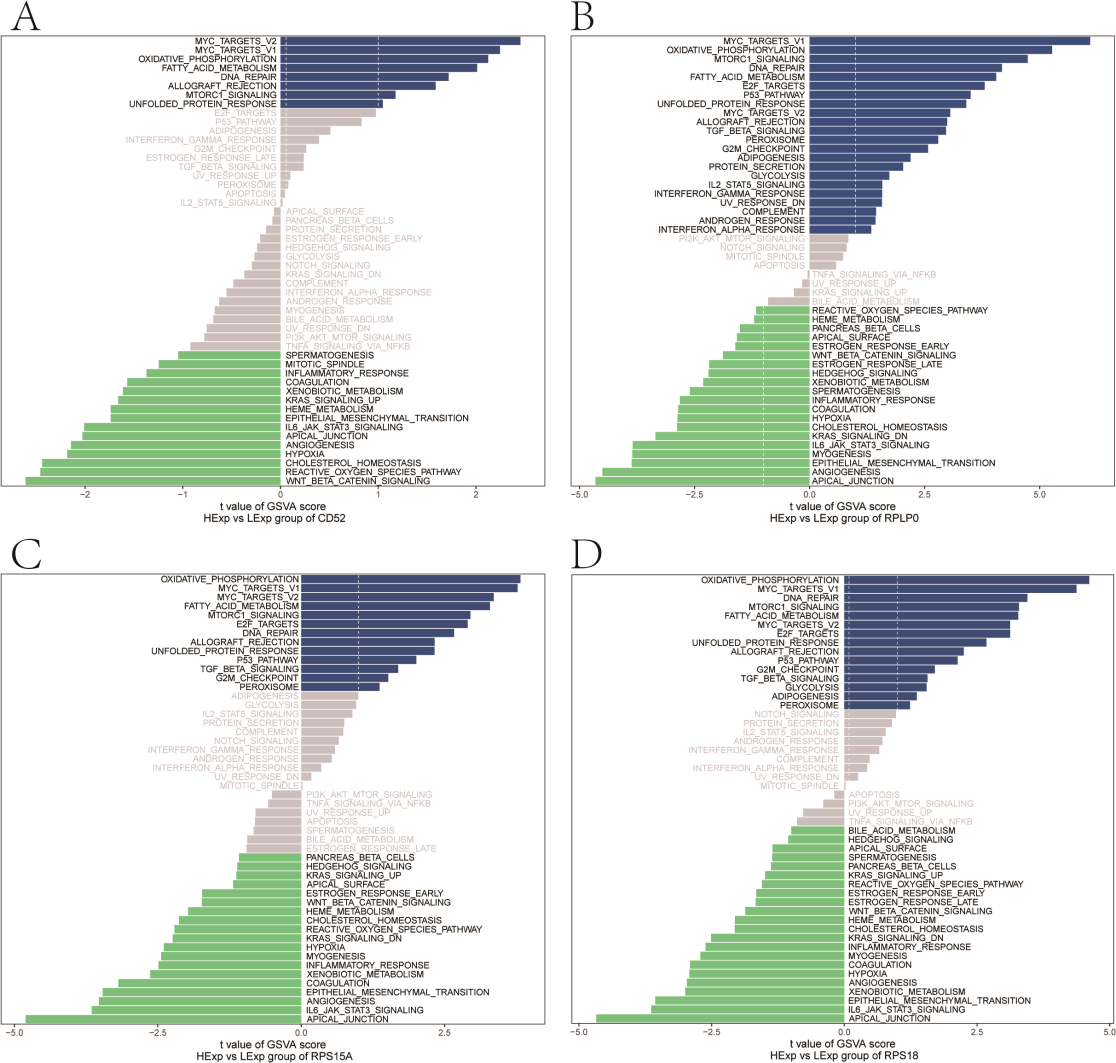
Gene Set Variation Analysis (GSVA) of the candidate genes. **(A–D)** GSVA results showing the signaling pathways associated with candidate gene expression. Blue represents signaling pathways enriched in the high-expression group, while green represents pathways enriched in the low-expression group. The analysis was conducted using the MSigDB Hallmark gene set as the reference background.

GSVA results showed that the pathways enriched by CD52 included MYC_TARGETS_V2, MYC_TARGETS_V1, OXIDATIVE_PHOSPHORYLATION and other pathways (**Figure 9A**); the pathways enriched by RPLP0 include MYC_TARGETS_V1, OXIDATIVE_PHOSPHORYLATION, TORC1_SIGNALING and other pathways (**Figure 9B**); the pathways enriched by RPS15A There are MTORC1_SIGNALING, P53_PATHWAY, TGF_BETA_SIGNALING and other pathways (**Figure 9C**); the pathways enriched by RPS18 include DNA_REPAIR, MTORC1_SIGNALING, TGF_BETA_SIGNALING and other pathways (**Figure 9D**). This suggests that key genes may influence disease progression through these pathways.

### 3.8 Expression profile of key genes in single cell data

**Figure 10:**
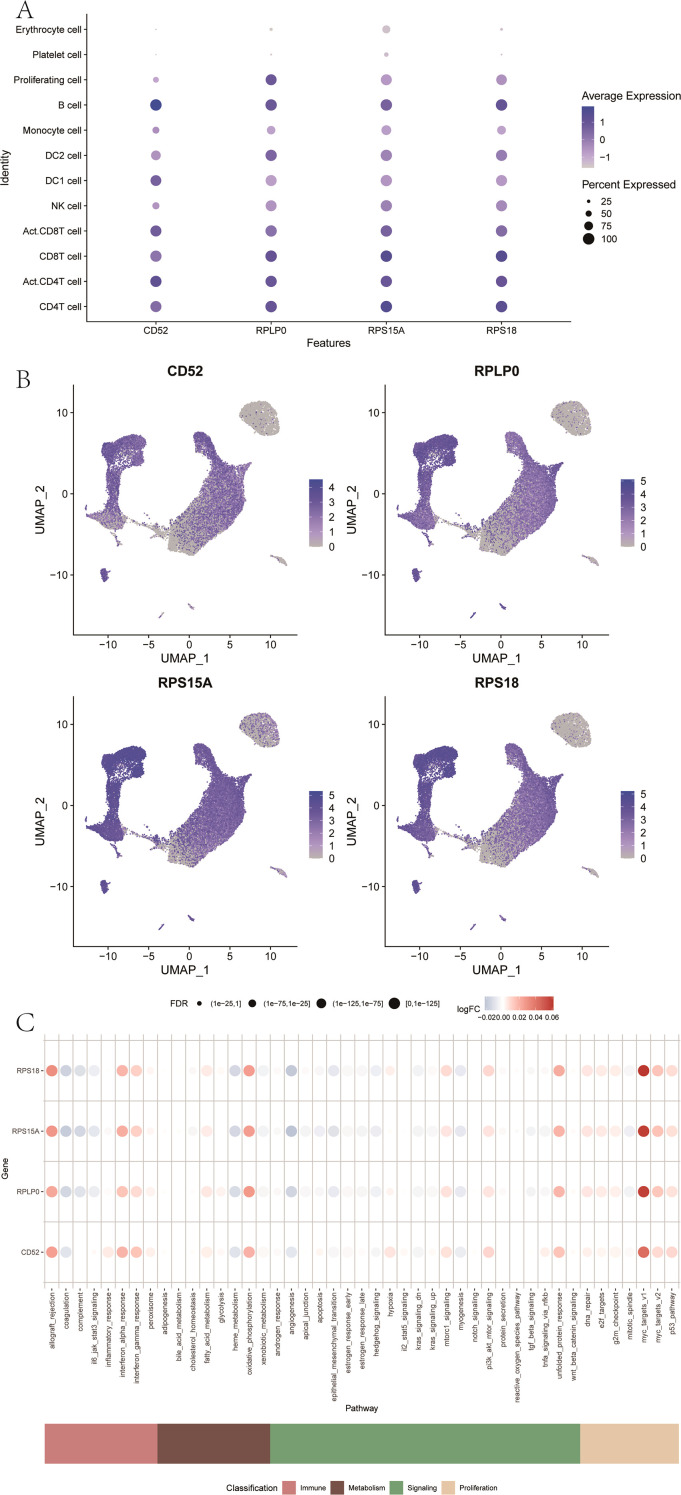
Expression profiles of candidate genes in single cells and their association with immunometabolic activity. **(A)** Dot plot illustrating the expression profiles of the candidate genes across various cell clusters. The size of each dot represents the percentage of cells expressing the gene, while the color gradient from gray to blue indicates the average expression level (with blue representing higher expression). **(B)** Feature plots (scatter plots) visualizing the expression of candidate genes at the single-cell level. Each dot represents an individual cell, with the color intensity reflecting the magnitude of gene expression within the UMAP/t-SNE space. **(C)** Activity differences of immunometabolic pathways associated with the candidate genes. The heatmap (or plot) displays the variation in metabolic pathway scores, where blue and red represent lower and higher activity levels, respectively.

We analyzed the expression of key genes in single cells and showed that key genes are expressed in CD4T cell, Act.CD4T cell, CD8T cell, Act.CD8T cell, NK cell, DC1 cell, DC2 cell, Monocyte cell, B cell, Expression in Proliferating cells, Platelet cells, and Erythrocyte cells (**Figures 10A, B**). In addition, we downloaded sepsis-related regulatory genes from the gene card database, analyzed the co-expression of key genes and disease-regulated genes in single cells, and displayed the top 3 genes among them (**Supplementary Figures 2–4**). Finally, this study used the AUCell function to calculate the enrichment scores of different immune/metabolic pathways for each cell. Then, the cells were divided into two groups based on the median expression of CD52, RPLP0, RPS15A, and RPS18, and the two groups were analyzed. Differences in enrichment scores of different immune, metabolic and other pathways (**Figure 10C**). This reveals the functional characteristics of four key genes in different cells and provides new clues for in-depth understanding of the interaction of immune and metabolic pathways at the single-cell level.

### 3.9 Validation of key gene expression in clinical samples

To validate our findings from single-cell RNA sequencing (scRNA-seq) and Mendelian randomization (MR), we performed qPCR on peripheral blood samples from sepsis patients (n=5) and healthy controls (n=5). The analysis confirmed that RPLP0 was significantly upregulated in the sepsis group (approx. 1.8-fold, p < 0.01). Conversely, CD52, RPS15A, and RPS18 were significantly downregulated, with expression reduced to 0.6-fold, 0.7-fold, and 0.5-fold of control levels, respectively (all p < 0.01). These findings provide direct experimental validation for the differential expression identified in activated CD4+ T cells (via scRNA-seq) and support the causal roles predicted by MR. These preliminary results are directionally consistent with the identification of RPLP0 as a potential risk gene and CD52, RPS15A, and RPS18 as potential protective genes, though they require validation in larger, clinically well-characterized cohorts (**Supplementary Figure 5**).

## 4 Discussion

### 4.1 Addressing the sepsis stratification challenge: an innovative framework based on causal inference

Sepsis, a heterogeneous syndrome defined by a dysregulated host response to infection leading to life-threatening organ dysfunction, constitutes a major global public health burden (Evans et al., 2021). Clinically, its heterogeneity manifests as diverse phenotypes ranging from mild infection to rapidly progressing septic shock and multi-organ failure, presenting immense challenges for diagnosis, treatment, and prognosis (Kumar, 2020). Although traditional biomarkers like procalcitonin (PCT) and C-reactive protein (CRP) are widely used, their specificity and sensitivity for distinguishing bacterial infections from non-infectious inflammation, assessing disease severity, or accurately predicting patient outcomes are insufficient (Pierrakos et al., 2020; Zhang et al., 2022). As highlighted in several recent high-level reviews, the sepsis field faces a pressing, unmet clinical need: the development of novel, mechanism-based biomarkers to achieve precise patient risk stratification and guide individualized treatment decisions (Sun et al., 2025).

The core innovation of this study lies in adopting an orthogonal, multi-omics, two-step strategy to move beyond traditional “correlation” studies and explore the “causal” drivers of sepsis immunopathology (Yu et al., 2025). First, using single-cell RNA sequencing (scRNA-seq), this study successfully pinpointed the pathological core of sepsis from the macroscopic tissue level to a specific cell subset—activated CD4^+^ T cells (Act.CD4T)—a critical cellular origin often obscured in traditional bulk RNA-seq analyses. While the expansion of activated CD4^+^ T cells in sepsis has been previously reported (e.g., Flynn et al., 2025) (Flynn et al., 2025), our study utilizes this subpopulation as a starting point to bridge the gap between cellular association and genetic causation through Mendelian Randomization. It should be noted that the central role of Act.CD4T cells in this context remains a plausible hypothesis derived from our integrated scoring system. Second, this study employed Mendelian Randomization (MR) analysis, using genetic variation as instrumental variables, to perform causal inference on 81 signature genes within Act.CD4T (Yasumizu et al., 2024). MR analysis simulates a “natural randomized controlled trial,” thereby identifying genes with a true causal effect on sepsis risk from within complex biological networks, rather than those merely reacting passively to the disease state (Zheng et al., 2023). We have innovatively integrated single-cell RNA sequencing and Mendelian randomization to investigate immunoregulatory gene mechanisms in sepsis. This strategy ultimately identified a causal signature panel of four genes: RPLP0 (Ribosomal Protein Large P0) was identified as a risk gene, while CD52, RPS15A (Ribosomal Protein S15a), and RPS18 (Ribosomal Protein S18) were identified as protective genes. More importantly, the functional enrichment analysis of these genes pointed to a unified biological concept: a previously under-appreciated regulatory axis in sepsis pathogenesis—the “Metabolism–Proteostasis–Immunity” axis (Costa-Mattioli and Walter, 2020; Kim et al., 2024). This trajectory-based approach reveals that the four-gene signature follows a coordinated expression program along the pseudotime continuum, suggesting these genes are early molecular drivers rather than late-stage bystanders in the collapse of the ‘Metabolism–Proteostasis–Immunity’ axis.

### 4.2 The four-gene signature panel: a new tool for clinical prognosis and precision stratification in sepsis

Translating complex bioinformatics discoveries into clinically actionable tools is the central goal of translational medicine. The four-gene signature panel (RPLP0, CD52, RPS15A, RPS18) identified in this study shows immense potential for clinical application, especially in the context of sepsis biomarkers shifting from single indicators to multi-gene expression profiles and machine learning models. Recent cutting-edge research has confirmed the clinical value of this trend (Du et al., 2024; Gao et al., 2025). For example, one study developed a machine learning model called “SepxFindeR,” based on a 6-gene hub panel (6-HubGss), capable of accurately distinguishing sepsis from septic shock; this panel’s detection is compatible with rapid RT-qPCR technology, highlighting its high translational potential (Du et al., 2024). Another study proposed a 4-gene panel (including TBX21, etc.) to assess the immune status of sepsis patients to guide immunotherapy decisions (Gao et al., 2025).

However, the four-gene panel proposed in this study provides a mechanistic complement to existing correlation-based models by incorporating genetically-supported causal inference: the depth of its biological basis and the certainty of its causal relationships. Most current prognostic models are constructed by screening high-dimensional transcriptomic data using machine learning algorithms; while they have predictive value, their biological mechanisms are often a “black box,” and they cannot distinguish causation from correlation. In contrast, our four-gene panel has two major advantages: 1. Cell-Specific: The panel is not derived from heterogeneous whole blood or tissue samples, but was refined from the core cell subset (Act.CD4T) involved in sepsis pathological progression, via scRNA-seq technology (Qiu et al., 2021). 2. Genetically Causal: Every gene in this panel has passed rigorous MR analysis, confirming a genetically causal link between its expression level and sepsis risk (Bai et al., 2025; Shi et al., 2025).

Therefore, this four-gene panel is not just a new prognostic biomarker, but more importantly, it is a mechanistically-defined endotype marker. This mechanistic insight is something that traditional SOFA or APACHE II scoring systems cannot provide. Based on this, we propose that this four-gene panel has clear clinical application prospects in the future intensive care unit. First, for severity grading and prognostic assessment, this study has already validated the differential expression of these four genes in the peripheral blood of a small clinical cohort (qPCR data) (RPLP0 upregulated, protective genes downregulated). In the future, RT-qPCR technology could be used to detect the expression profile of this panel in peripheral blood to construct a quantitative ‘risk score’. This score holds promise for early warning of sepsis, severity grading (e.g., dynamic comparison with SOFA scores), and as an independent indicator for predicting 28-day mortality or risk of progression to septic shock (similar to the function of the SepxFindeR model (Du et al., 2024)). Second, for patient stratification for precision therapy, this is the panel’s most valuable translational application. It can stratify sepsis patients who have similar clinical phenotypes but different underlying pathological mechanisms (endotypes) to guide clinical trial design and individualized treatment. For example, patients with high RPLP0 expression (“high-risk type”) may represent an endotype dominated by proteostasis imbalance and a HIF-1-driven inflammatory storm, and should be prioritized for enrollment in clinical trials for RPLP0 inhibitors or HIF-1/IL-17 pathway blockers (Zhang et al., 2024). Conversely, patients with low RPS15A/RPS18 expression (“low-protection type”) may represent an endotype characterized by immunometabolic failure and AMPK pathway inhibition, and might be more suitable for metabolic regulatory therapies such as AMPK agonists (e.g., metformin). While elevated biomarkers are traditionally favored in clinical settings, the downregulation of protective genes (CD52, RPS15A, RPS18) serves as a critical indicator of ‘protective deficiency’ endotypes. In the era of precision medicine, quantifying this loss of homeostatic capacity through RT-qPCR allows for the identification of patients who may benefit from compensatory therapies, such as AMPK agonists, to restore the ‘Metabolism–Proteostasis–Immunity’ balance.

### 4.3 Mechanistic deep dive: a “Metabolism–Proteostasis–Immunity” regulatory axis

This study identified RPLP0 (a ribosomal protein) as a core causal risk gene for sepsis through MR analysis, suggesting it is not merely a result of the disease but a key node driving its progression. Through GSEA and GSVA pathway enrichment analysis, this study’s (draft) discussion further revealed that RPLP0 ‘s pathogenic effect may be realized through a multi-dimensional pathological network that integrates the IL-17 signaling pathway, the HIF-1 signaling pathway, and proteostasis (e.g., Endoplasmic Reticulum Stress, ERS) pathways (Liu et al., 2025). This discovery is of great clinical significance, positioning RPLP0 as a multimodal pathological hub connecting three core pillars of sepsis pathology: 1. Proteostasis Collapse: As a component of the large ribosomal subunit, abnormal RPLP0 expression (upregulated in this study) may directly interfere with ribosome biogenesis and function, leading to errors in protein translation and folding defects, thereby triggering intense Endoplasmic Reticulum Stress (ERS). Our study’s discussion has already pointed out that RPLP0 overexpression can activate the IRE1α-XBP1 axis via the ERS pathway, thereby driving NLRP3 inflammasome assembly and IL-1β release (Wang et al., 2024). 2. Hypoxic Maladaptation: GSEA analysis showed RPLP0 is enriched in the HIF-1 signaling pathway. HIF-1α (Hypoxia-inducible factor-1α) has been confirmed by multiple authoritative 2024 reviews as a core regulator in sepsis pathogenesis and progression. It connects tissue hypoxia, dysregulated inflammatory responses, immune cell dysfunction, and ultimately, organ failure (Liu et al., 2025). The causal association between RPLP0 and HIF-1 suggests that high RPLP0 expression may amplify the HIF-1 signal, exacerbating the body’s pathological hypoxic response and metabolic disturbance under the low-perfusion state of sepsis, thus promoting organ dysfunction. 3. Excessive Inflammatory Response: RPLP0 drives the “cytokine storm” through at least two pathways. First, as mentioned, via the “ERS-NLRP3” axis to release IL-1β (Xu and Núñez, 2023); second, this study’s GSEA analysis clearly showed its enrichment with the IL-17 signaling pathway. IL-17 is a potent pro-inflammatory cytokine that has been confirmed as a key mediator in the sepsis inflammatory response (Ge et al., 2020; Li et al., 2020). In summary, high RPLP0 expression may define a specific, highly malignant sepsis endotype: the “Proteostasis Imbalance–Hypoxic Maladaptation–High Inflammation” type. This finding offers dual value to the clinic: RPLP0 expression levels can serve as a diagnostic biomarker for this specific endotype; simultaneously, RPLP0 itself, along with its downstream HIF-1 and IL-17 pathways, constitutes highly attractive, causally-supported targets for precision therapy.

In contrast to the risk effect of RPLP0, the protective gene cluster discovered in this study (CD52, RPS15A, RPS18) shares a common feature: their central role in immunometabolism and maintaining proteostasis. This study’s functional enrichment analysis showed CD52 is related to oxidative phosphorylation (OXPHOS) (Qiu et al., 2021), while the two protective ribosomal proteins (RPS15A, RPS18) are closely linked to the AMPK and mTORC1 signaling pathways (Yumoto and Coopersmith, 2024). This strongly suggests that maintaining the metabolic health and proteostasis of cells (especially Act.CD4T) is a key protective mechanism against sepsis progression. This discovery forms a perfect mechanistic loop with major breakthroughs in the fields of immunometabolism and ribosomal dysfunction from the last two years (2024-2025): (1) Protective Role of the AMPK Pathway: This study found the protective genes are related to the AMPK pathway. An authoritative 2024 review highlights the potential of AMPK activation as a therapeutic target in sepsis (Yumoto and Coopersmith, 2024). As a cellular energy sensor, AMPK activation has multiple protective effects, including anti-inflammation, promoting mitochondrial biogenesis, inducing autophagy, and (critically) overcoming the “immunometabolic paralysis” state (Yumoto and Coopersmith, 2024). Furthermore, a key downstream effect of AMPK is the inhibition of the mTORC1 signaling pathway (Darweesh et al., 2025). (2) mTORC1-Mediated Pathological Ribophagy: This study found that high expression of the ribosomal proteins RPS15A and RPS18 is protective. The profound meaning of this finding was revealed by a groundbreaking 2025 study, which found that during sepsis, platelets undergo an mTORC1-dependent ribophagy—degrading ribosomes to release amino acids, providing fuel for aerobic glycolysis, thereby maintaining pathological platelet hyperactivity and thrombosis (Bandala-Sanchez et al., 2018). That study specifically identified RPS15A as one of the key substrates in this pathological degradation process. (3) Mechanism Integration: The aforementioned evidence chain perfectly explains this study’s findings. Our MR analysis shows that having (high expression of) RPS15A/RPS18 is protective; the 2025 study shows that losing (degrading) RPS15A is pathogeni (Bandala-Sanchez et al., 2018). Connecting these two is the signaling pathway: our protective genes are associated with the (protective) AMPK pathway; AMPK exerts its effect by inhibiting mTORC1 (Darweesh et al., 2025); and mTORC1 is the upstream signal driving (pathogenic) ribophagy (degradation of RPS15A).

Therefore, the protective gene signature defined by this study (high RPS15A/RPS18 and high CD52) defines a patient subset capable of maintaining immunometabolic homeostasis (Bandala-Sanchez et al., 2018). By maintaining AMPK pathway activity, they inhibit mTORC1-mediated pathological ribosomal degradation, thereby protecting the integrity of proteostasis, and maintain mitochondrial metabolic health via the CD52 -associated OXPHOS pathway (Zarou et al., 2024). This discovery provides strong causal evidence supporting a treatment for sepsis: activating this protective axis may have therapeutic value. For example, AMPK agonists (such as metformin), which have been shown to have significant benefits in preclinical sepsis models are supported by our study with genetic and causal mechanisms for their use in humans. Furthermore, CD52 agonists (such as Alemtuzumab) represent a potential therapeutic avenue that could be explored for ‘repurposing’ to potentially reverse sepsis-related immunometabolic paralysis (Saraiva et al., 2024; Yumoto and Coopersmith, 2024).

### 4.4 Conclusion, limitations, and future prospects

By integrating scRNA-seq and MR analysis, this study provides a causal evidence chain linking sepsis risk to key genes within a specific cell subset (Act.CD4T). The “Metabolism–Proteostasis–Immunity” regulatory axis model we propose offers a new theoretical framework for understanding the heterogeneity of sepsis (Baechle et al., 2023). This model is not an abstract concept but a dynamic equilibrium state quantified and defined by the four-gene signature panel identified in this study: Metabolism is regulated by CD52 (OXPHOS) and RPS15A/RPS18 (AMPK); Proteostasis is determined by the antagonism between RPLP0 (ERS stress) and RPS15A/RPS18 (ribosomal stability); and Immunity is the final phenotype resulting from the imbalance or maintenance of the first two (high inflammation vs. immune homeostasis). This four-gene panel can thus be viewed as a “balance indicator” reflecting the immunometabolic state of Act.CD4T, offering unprecedented opportunities for the precision subtyping and treatment of sepsis. establishing the panel as a clinical prognostic tool by correlating it with hard clinical endpoints, such as SOFA, APACHE II scores and 28-day mortality.

Nevertheless, this study has some limitations. First, the eQTL and GWAS databases (like eQTLGen) relied upon by the MR analysis are predominantly based on European-ancestry populations (Liu et al., 2021). Whether these causal associations apply to other ethnicities (e.g., East Asian populations) requires further validation in multi-center, multi-ethnic cohort studies. Second, our qPCR validation cohort was limited (5 sepsis patients vs. 5 healthy controls). Critically, these results demonstrated perfect directional concordance with the causal effects predicted by our large-scale Mendelian Randomization analysis (n=486,484). This strong convergence from two orthogonal methodologies—genetic causal inference and clinical expression—provides a robust reciprocal validation that transcends the small sample size. Nevertheless, while this cohort validates the *causal direction*, a larger prospective study is required to establish the panel as a clinical prognostic tool by correlating it with hard clinical endpoints, such as SOFA, APACHE II scores and 28-day mortality. Third, a technical limitation of scRNA-seq is the capture bias against fragile cell types, particularly neutrophils, which are often underrepresented due to low mRNA content and high RNase activity. While we partially addressed this via CIBERSORT deconvolution to explore neutrophil-related correlations, their specific contribution to the ‘Metabolism–Proteostasis–Immunity’ axis may not be fully captured at the single-cell level. Fourth, the regulatory networks identified via RcisTarget are based on motif enrichment predictions. While this method offers systemic insights, it may include false-positive results; thus, future experimental validation, such as ChIP-seq or CUT&Tag, is warranted to definitively confirm the direct binding of these key TFs. Fifth, the findings of this study are all at the transcriptional (RNA) level; future work is needed to validate the expression and functional changes of the key proteins (RPLP0, CD52, etc.) in patient samples using proteomics techniques (such as Western Blot or ELISA) (Miao et al., 2021). Sixth, a discrepancy was noted between the scRNA-seq results and CIBERSORT deconvolution (**Figure 6C**), where the latter showed no significant change in CD4^+^ T cell proportions. This likely reflects the limitation of bulk deconvolution in resolving fine-grained activation states compared to high-resolution single-cell clustering.

Beyond technical constraints, it is essential to acknowledge the conceptual caveats of our multi-step analytical framework. While the integration of scRNA-seq and MR allows for the identification of causal drivers, the resulting four-gene signature represents a tentative deduction and should be viewed as a hypothesis-generating result rather than a definitive clinical answer. Notably, although our study transcends purely computational inference by providing preliminary clinical evidence via qPCR, the small sample size (n=5) and the absence of detailed clinical stratification—such as disease stage or microbial etiology (Gram-negative vs. Gram-positive)—limit the immediate generalizability of these findings. Sepsis is a highly dynamic and heterogeneous syndrome; therefore, this signature should be interpreted as a collection of ‘potential candidate biomarkers’. Their specific utility in patient stratification, severity assessment, or prognostic prediction remains to be rigorously interrogated in large-scale, prospective clinical cohorts that account for the full spectrum of sepsis diversity.

Looking ahead, this study opens three main directions for translational research in sepsis: First, the establishment and validation of a clinical prognostic model: launching a large-scale, prospective, multi-center clinical study using RT-qPCR to detect the four-gene panel’s expression profile in peripheral blood at the time of sepsis admission, to build and validate its predictive model for sepsis severity, progression to septic shock, and 28-day mortality. Second, preclinical intervention studies: systematically validating the therapeutic potential of this regulatory axis in sepsis animal models (e.g., CLP model). Specifically, testing the effects of RPLP0 inhibitors, AMPK agonists (like metformin) (Saraiva et al., 2024; Yumoto and Coopersmith, 2024), and CD52 agonists (like Alemtuzumab), alone or in combination, on sepsis survival rates and organ damage. Third, spatial-resolved pathological mechanisms: using emerging technologies like Spatial Transcriptomics to analyze the *in situ*, high-resolution spatial distribution and interaction of this four-gene panel and its downstream signals (e.g., HIF-1, mTORC1) in tissue sections of sepsis-induced MOF (e.g., lung, kidney), to visually confirm whether this “Metabolism–Proteostasis–Immunity” axis is functioning at the “storm center” of organ damage (Janosevic et al., 2021).

In conclusion, this study not only provides a new list of sepsis biomarkers but, more importantly, it offers an operable, causally-validated pathogenic framework. This four-gene signature panel and the “Metabolism–Proteostasis–Immunity” axis it represents provide a solid theoretical foundation and a clear translational path for developing urgently needed precision medicine strategies for sepsis.

## Data Availability

Publicly available datasets were analyzed in this study. Public datasets analyzed are available in GEO (GSE175453, GSE28750), eQTLGen (https://www.eqtlgen.org), and IEU OpenGWAS (ieu-b-4980). Clinical validation data are available from the corresponding author upon reasonable request.

## Supplementary material

Supplementary Figure 1 Single-cell Pre-processing **(A)** Single-cell quality control (QC) metrics, showing the total number of cells, gene counts, and sequencing depth for each sample. **(B)** Correlation between sequencing depth and mitochondrial content (left) and between sequencing depth and gene counts (right). The scatter plots display the relationship between mitochondrial content (y-axis) and nCount_RNA (x-axis). Each dot represents an individual cell, illustrating the distribution of RNA counts relative to mitochondrial gene expression levels. **(C)** Identification of genes with significant intercellular variation and the corresponding variable feature plot. **(D)** Scree plot displaying the ranking of variance explained by each Principal Component (PC). **(E, F)** Visualization of Principal Component Analysis (PCA) and the distribution of PCs, where dots represent individual cells and colors indicate different samples.

Supplementary Figures 2–4 Co-expression analysis of key genes in single cells. **(A-D)** Gene co-expression between disease-regulated genes and key genes in single-cell data, along with the correlation analysis of these co-expressed genes.

Supplementary Table 1 Clinical characteristics of subjects.

Supplementary Table 2 Primer sequences.

Supplementary Table 3 Identification and screening pipeline for causal genes in sepsis.
