# Supplementary figures and images for: A synergistic multi-omics approach: causal sepsis drivers identified in activated CD4+ T cells by single-cell RNA sequencing and Mendelian randomization
Source: Front Cell Infect Microbiol. 2026 May 28;16:1749207. doi: 10.3389/fcimb.2026.1749207 (PMC13253298; doi:10.3389/fcimb.2026.1749207)

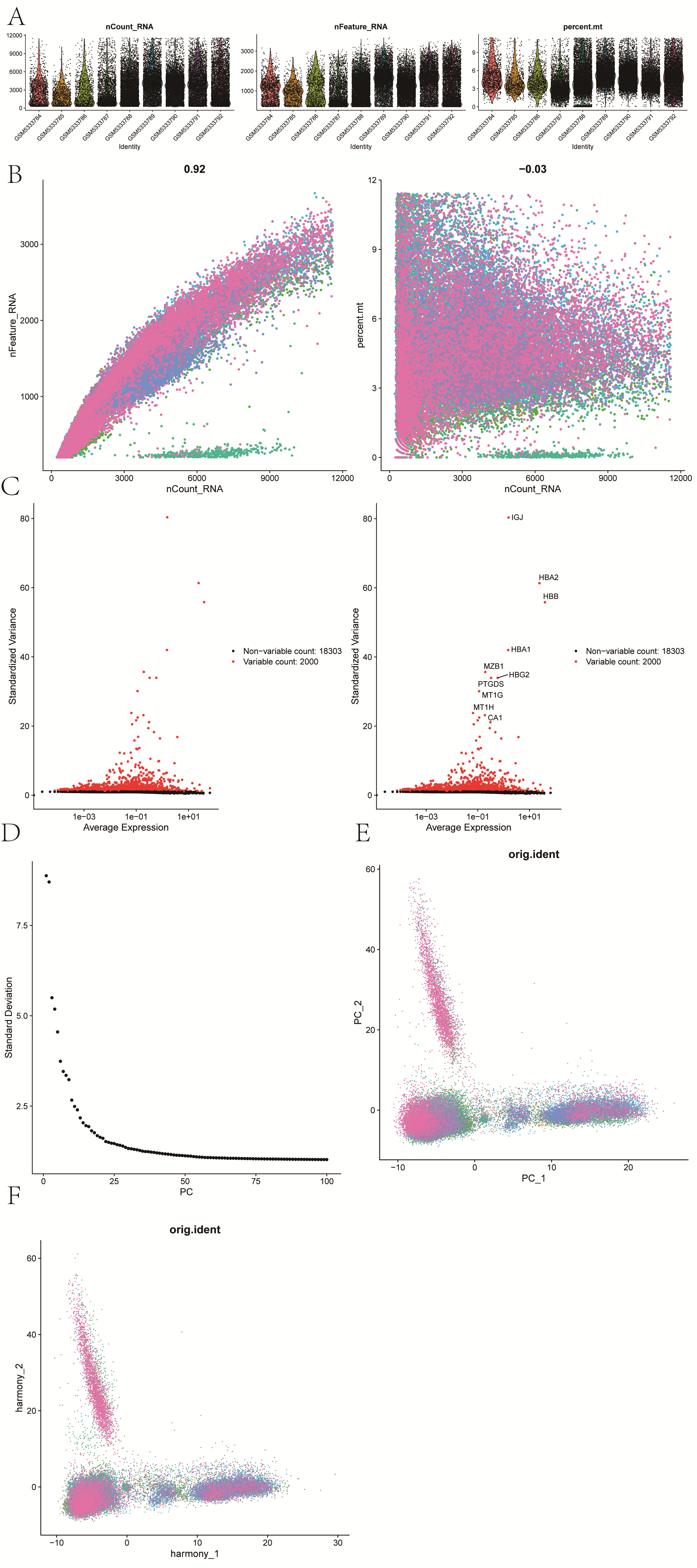

Supplement: Supplementary Figure 1 — Single-cell Pre-processing (A) Single-cell quality control (QC) metrics, showing the total number of cells, gene counts, and sequencing depth for each sample. (B) Correlation between sequencing depth and mitochondrial content (left) and between sequencing depth and gene counts (right). The scatter plots display the relationship between mitochondrial content (y-axis) and nCount_RNA (x-axis). Each dot represents an individual cell, illustrating the distribution of RNA counts relative to mitochondrial gene expression levels. (C) Identification of genes with significant intercellular variation and the corresponding variable feature plot. (D) Scree plot displaying the ranking of variance explained by each Principal Component (PC). (E, F) Visualization of Principal Component Analysis (PCA) and the distribution of PCs, where dots represent individual cells and colors indicate different samples. [file Image1.tif]

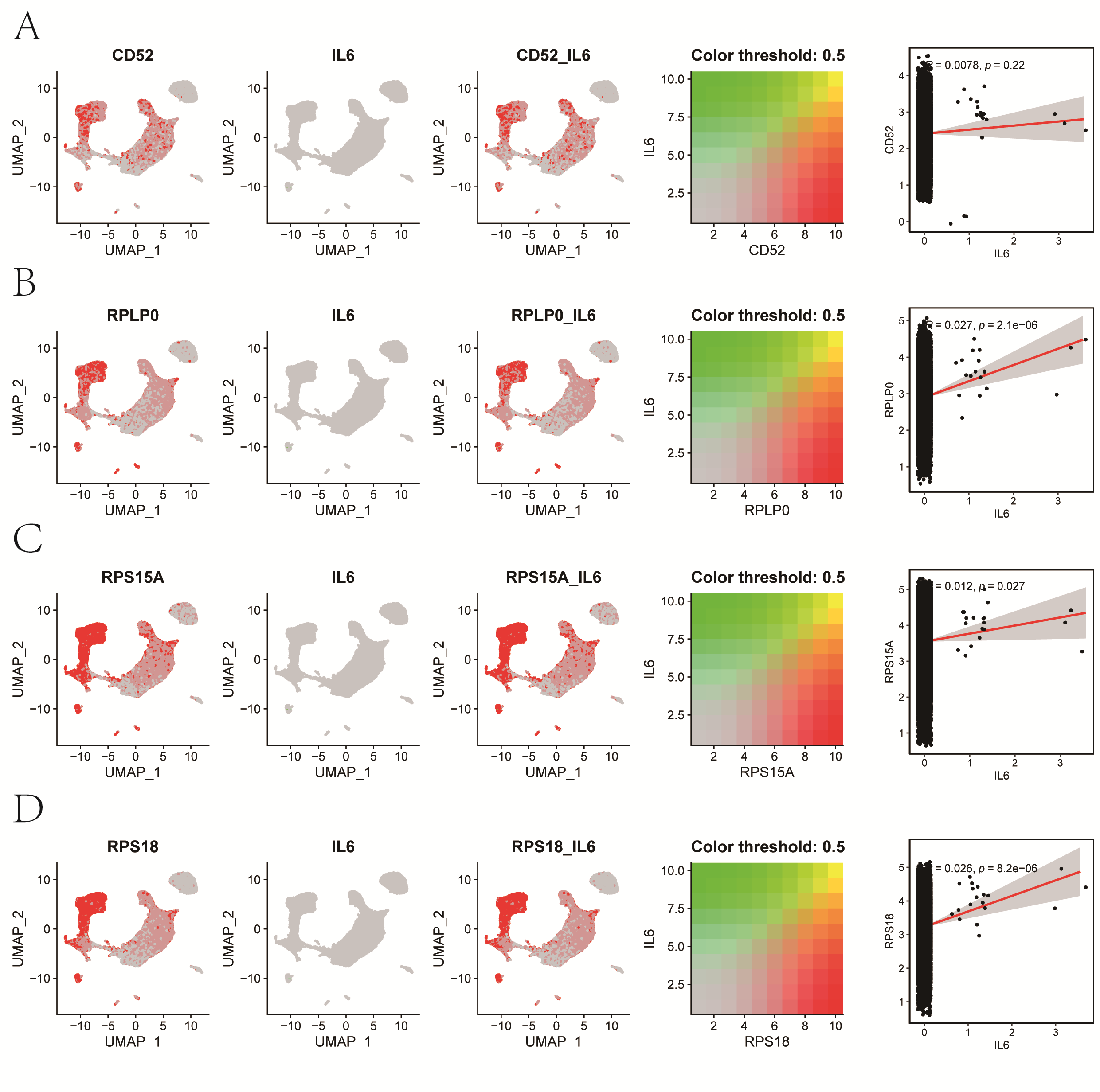

Supplement: Supplementary Figures 2–4 — Co-expression analysis of key genes in single cells. (A-D) Gene co-expression between disease-regulated genes and key genes in single-cell data, along with the correlation analysis of these co-expressed genes. [file Image2.tif]

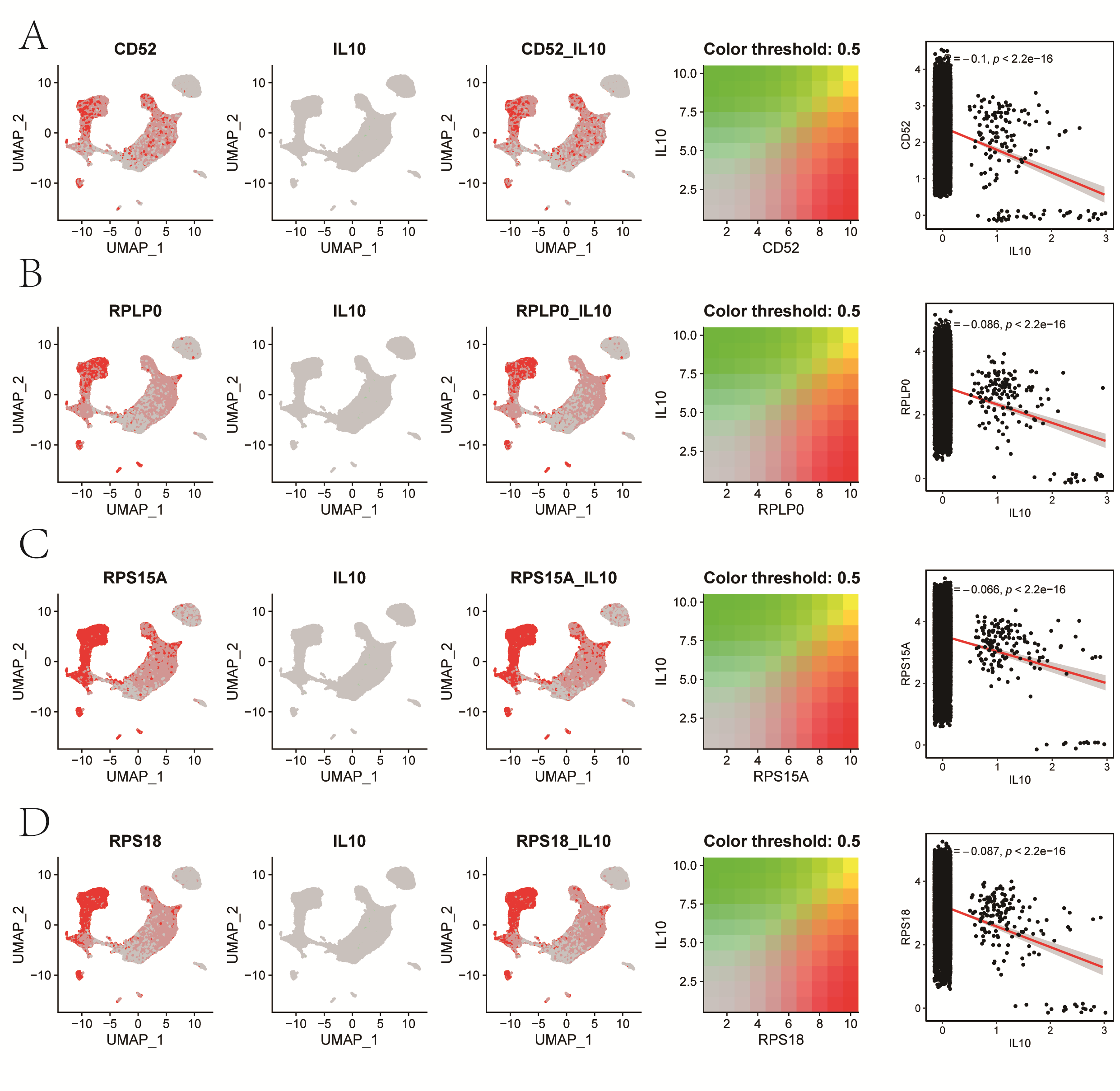

Supplement: Supplementary file 3 [file Image3.tif]

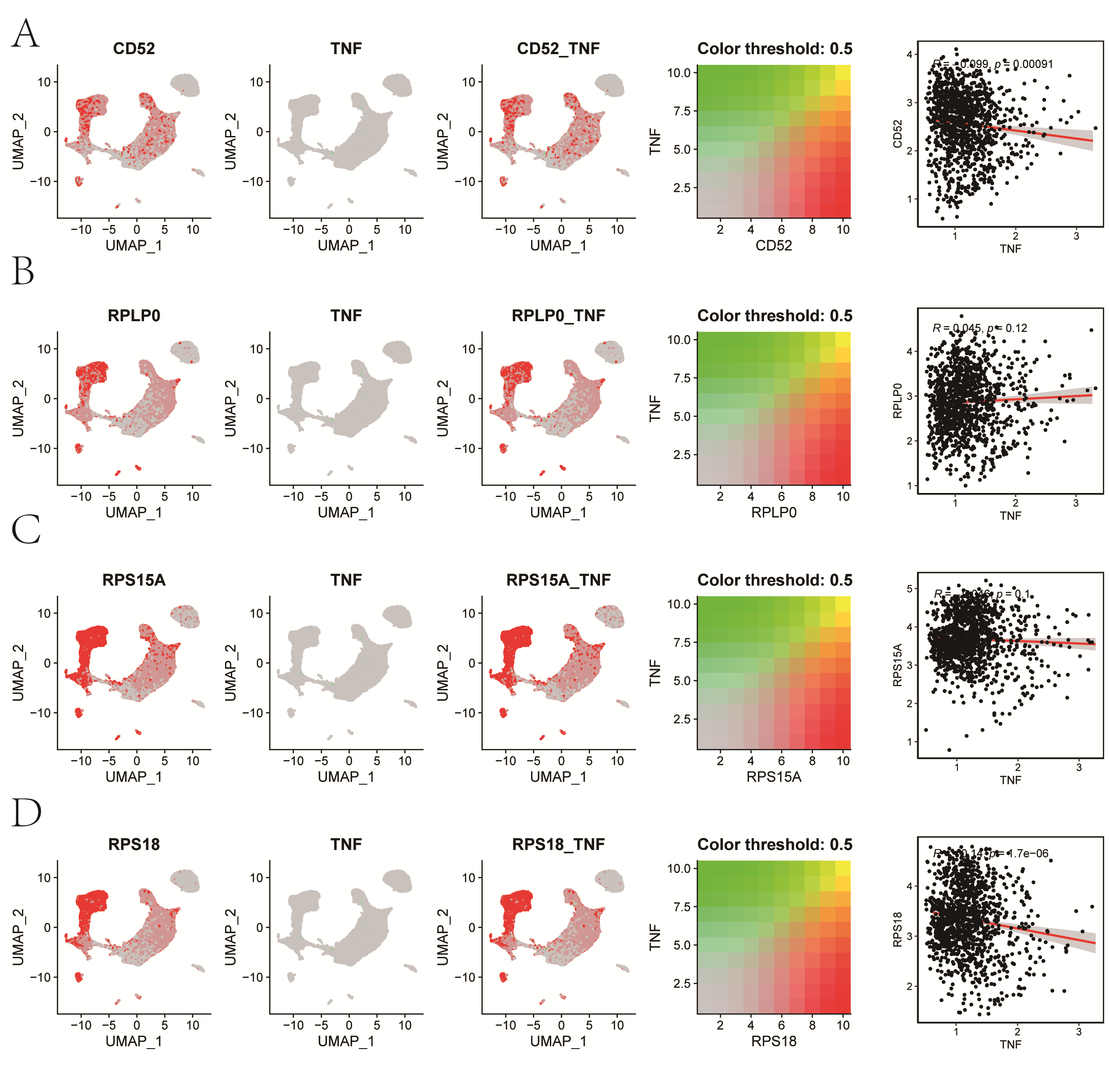

Supplement: Supplementary file 4 [file Image4.tif]
