# Supplementary material for: A synergistic multi-omics approach: causal sepsis drivers identified in activated CD4+ T cells by single-cell RNA sequencing and Mendelian randomization
Source: Front Cell Infect Microbiol. 2026 May 28;16:1749207. doi: 10.3389/fcimb.2026.1749207 (PMC13253298; doi:10.3389/fcimb.2026.1749207)
Supplement: Supplementary file 8 [file Presentation1.pptx]

## Slide 1
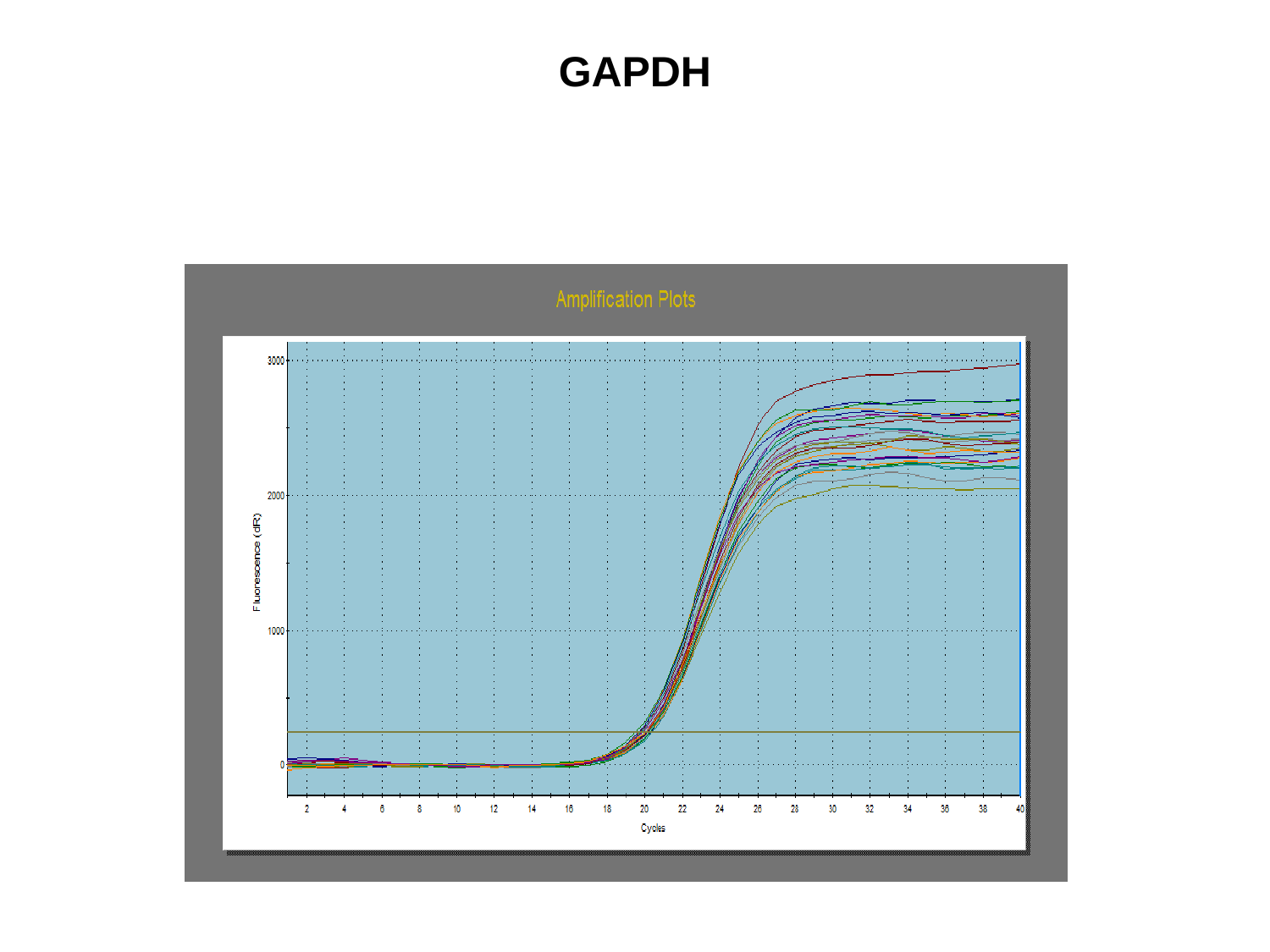

#
GAPDH

## Slide 2
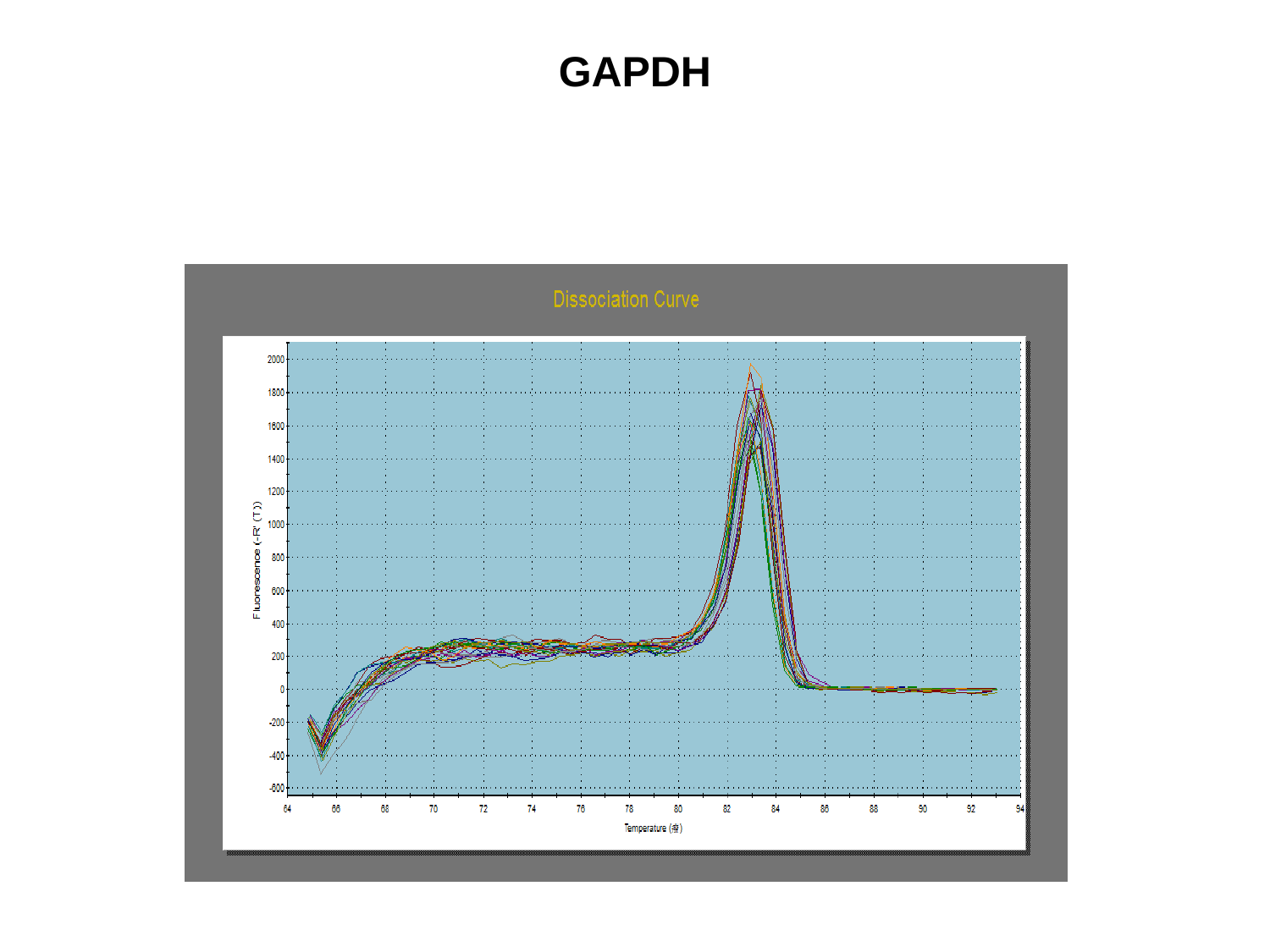

#
GAPDH

## Slide 3
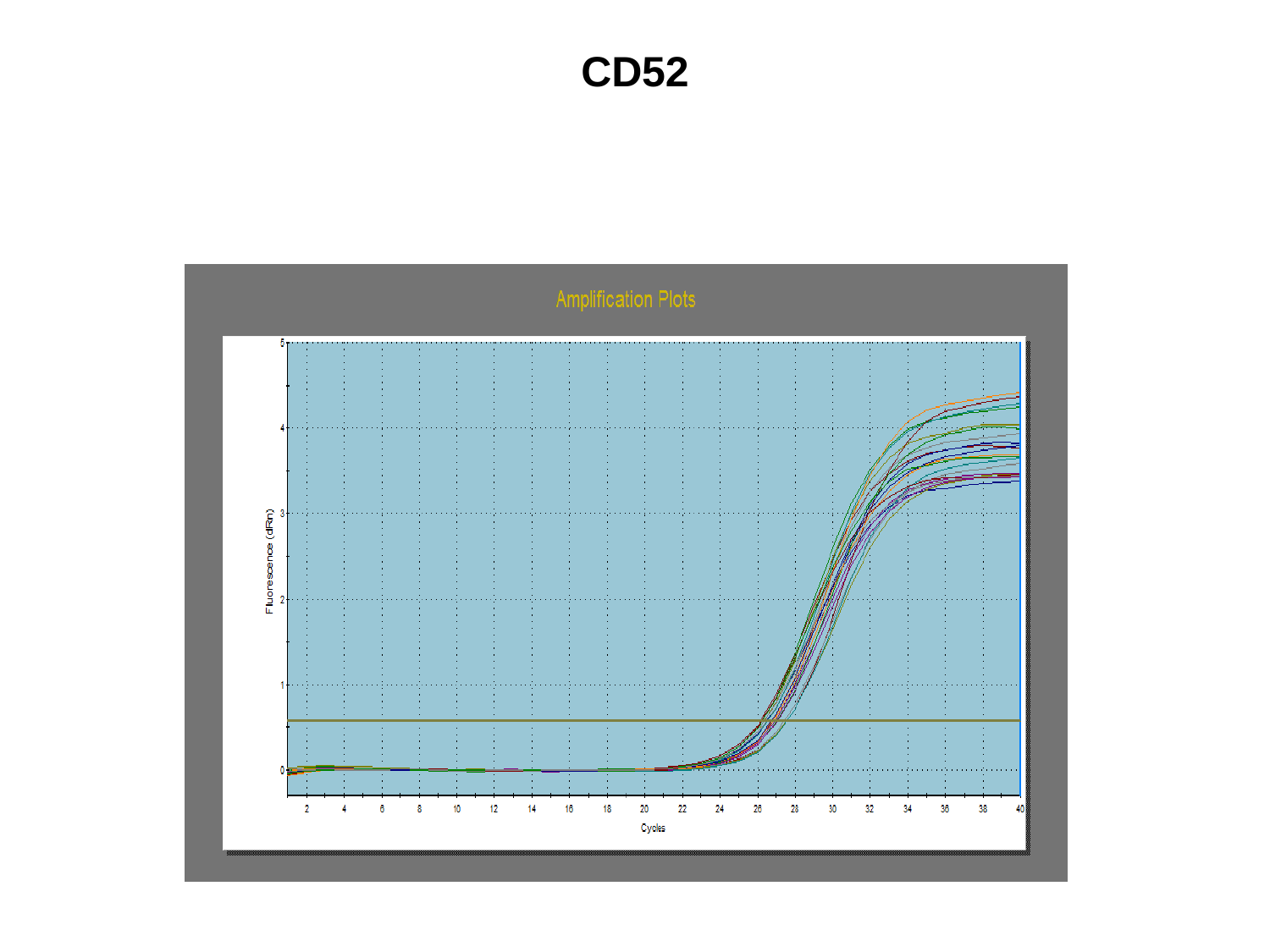

#
CD52

## Slide 4
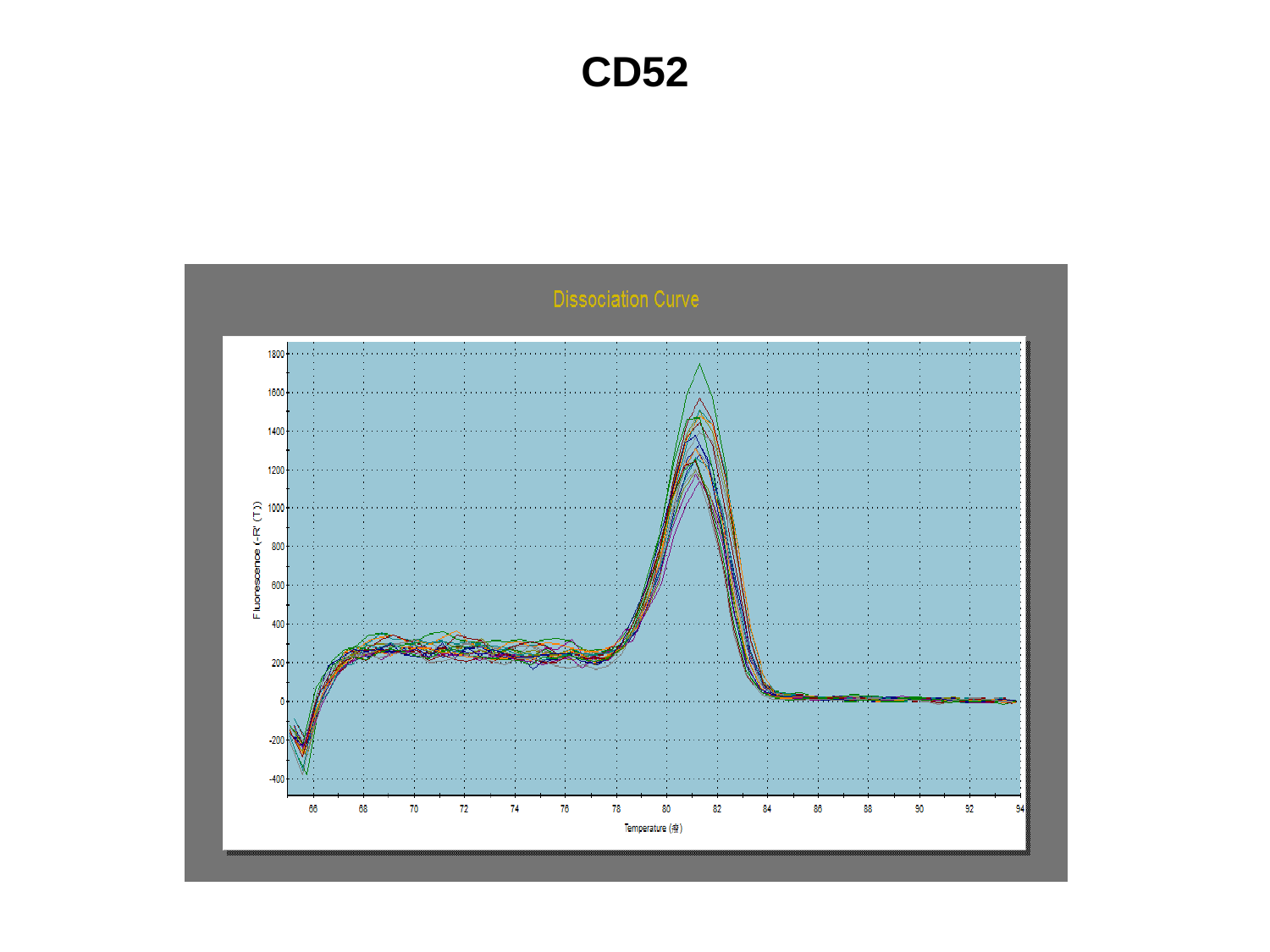

#
CD52

## Slide 5
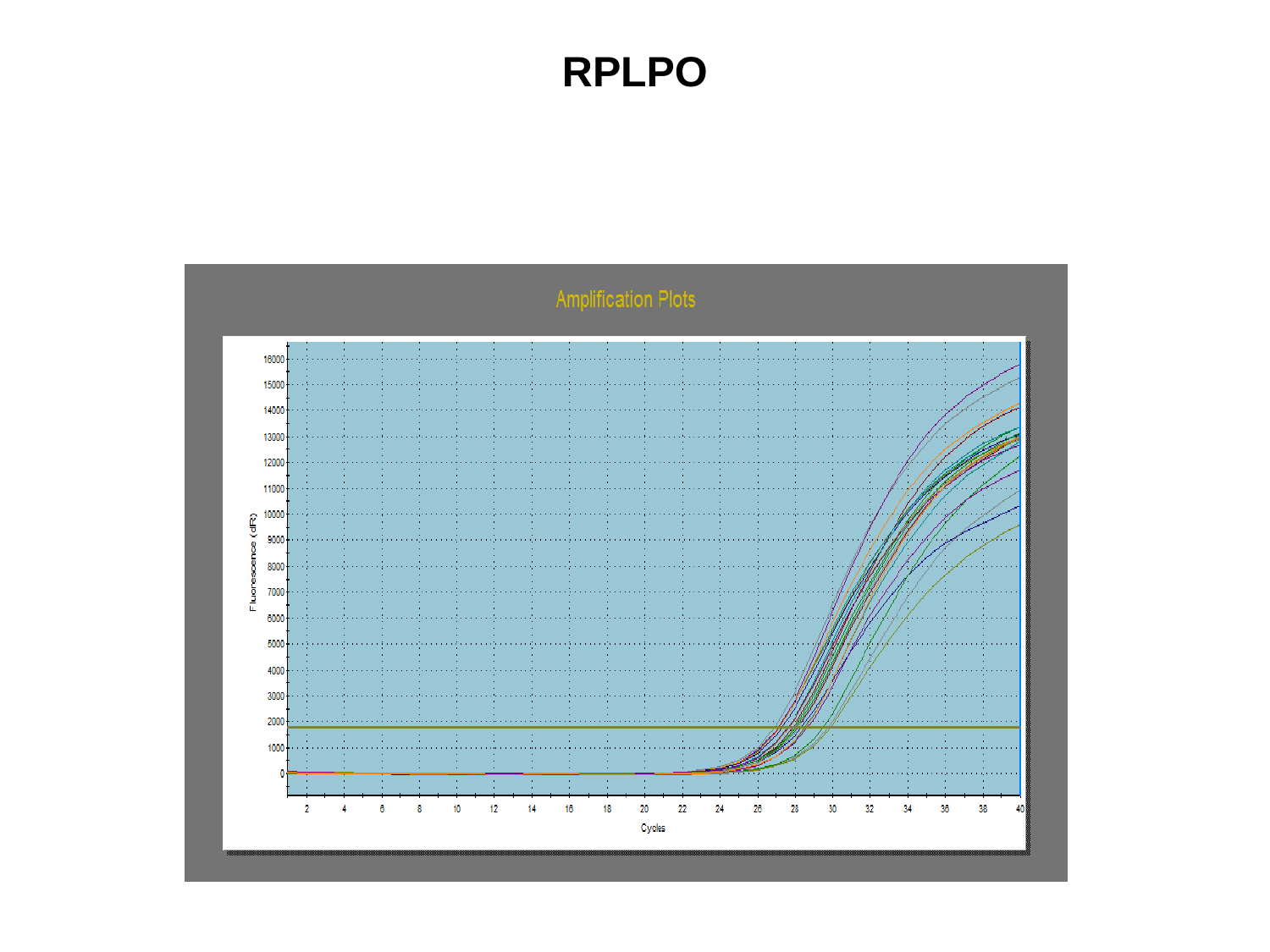

#
RPLPO

## Slide 6
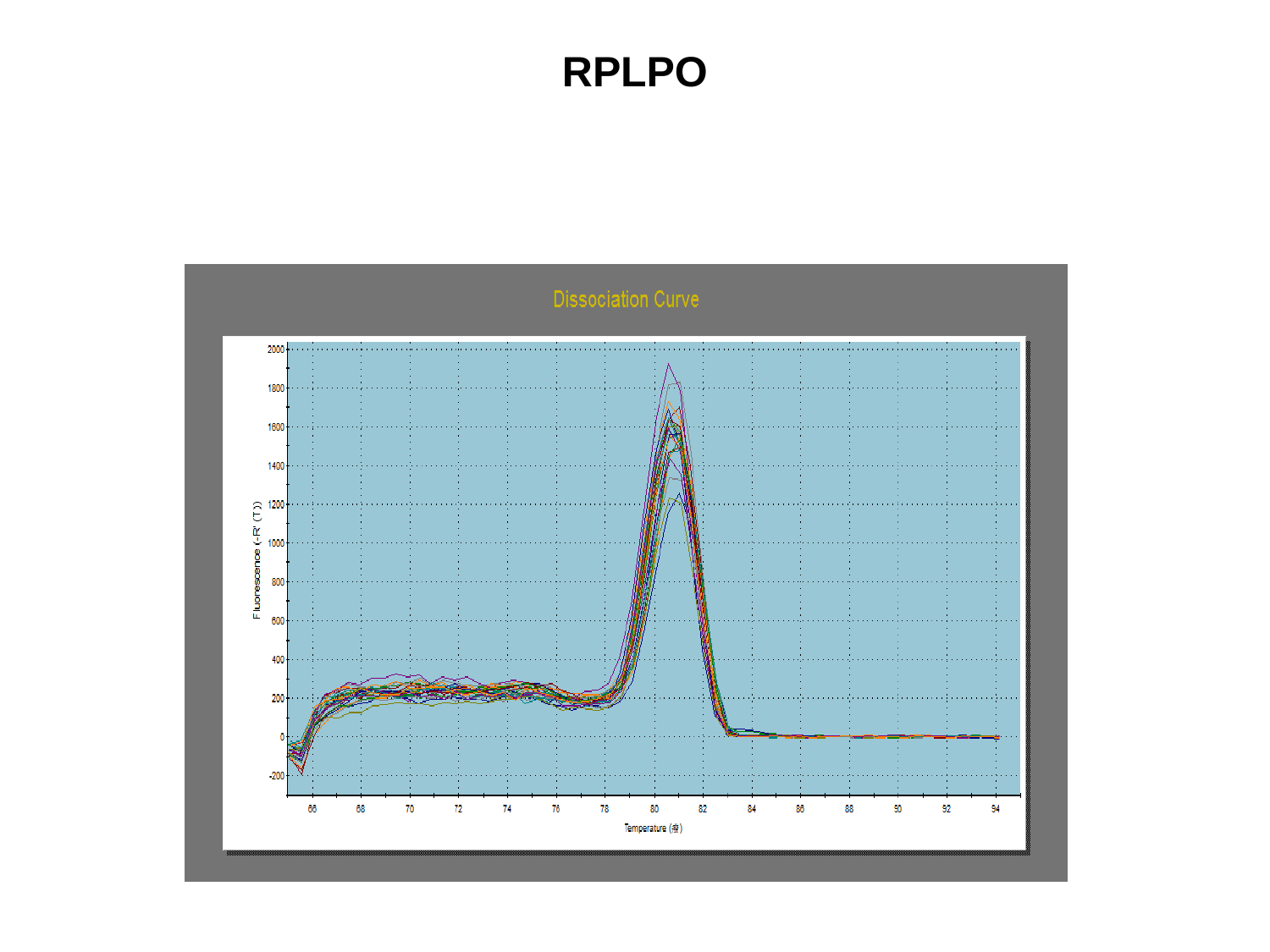

#
RPLPO

## Slide 7
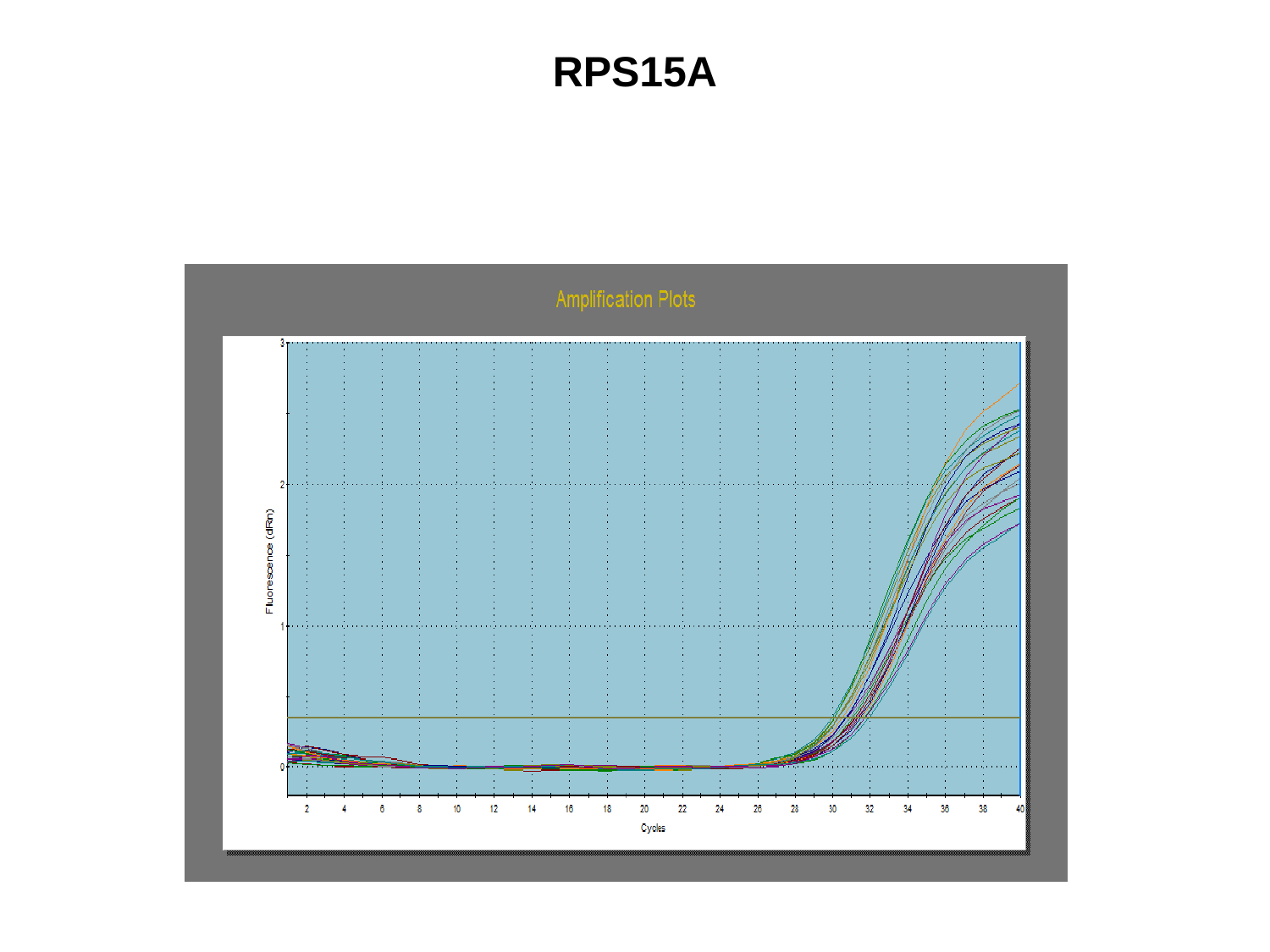

#
RPS15A

## Slide 8
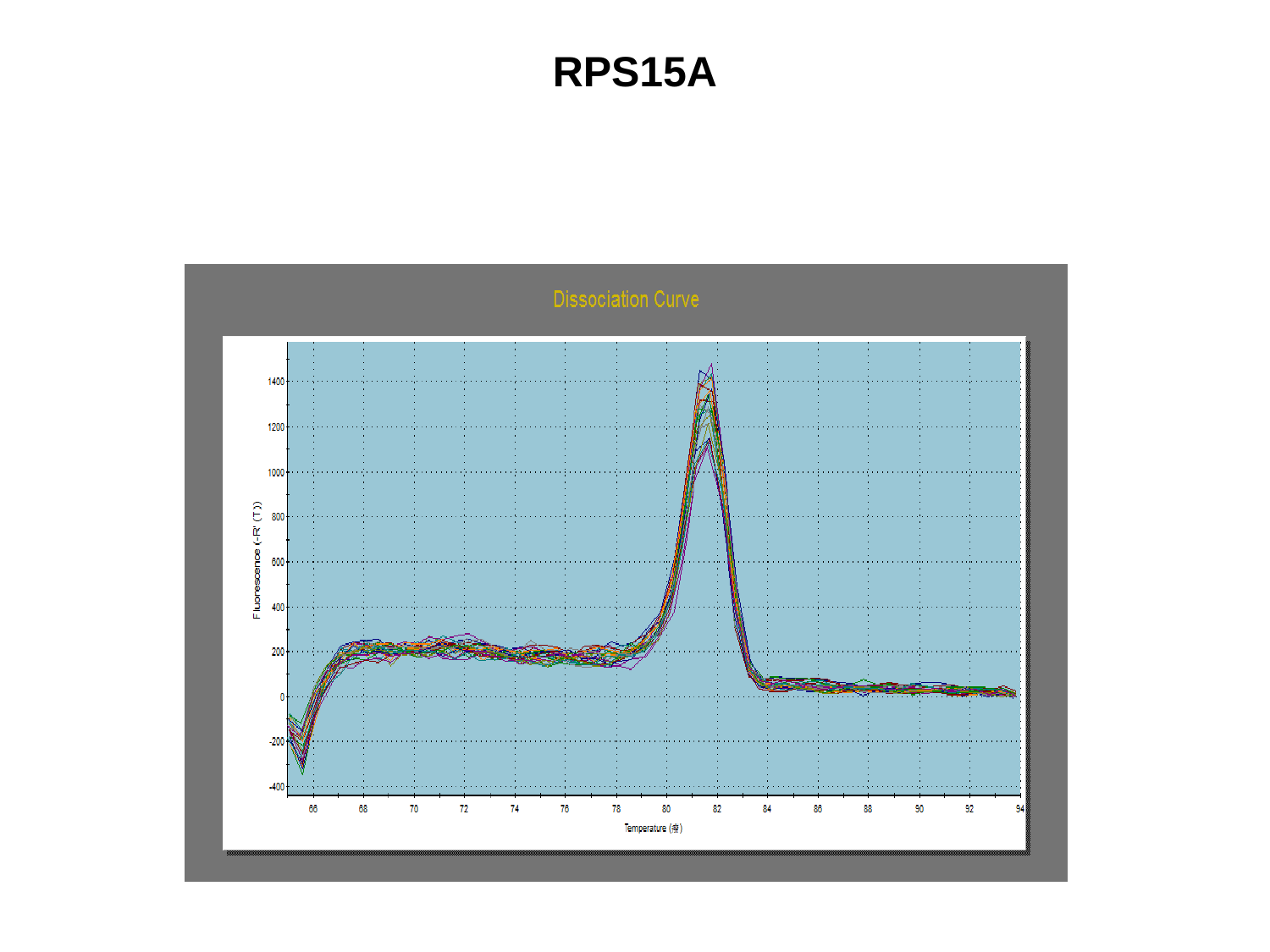

#
RPS15A

## Slide 9
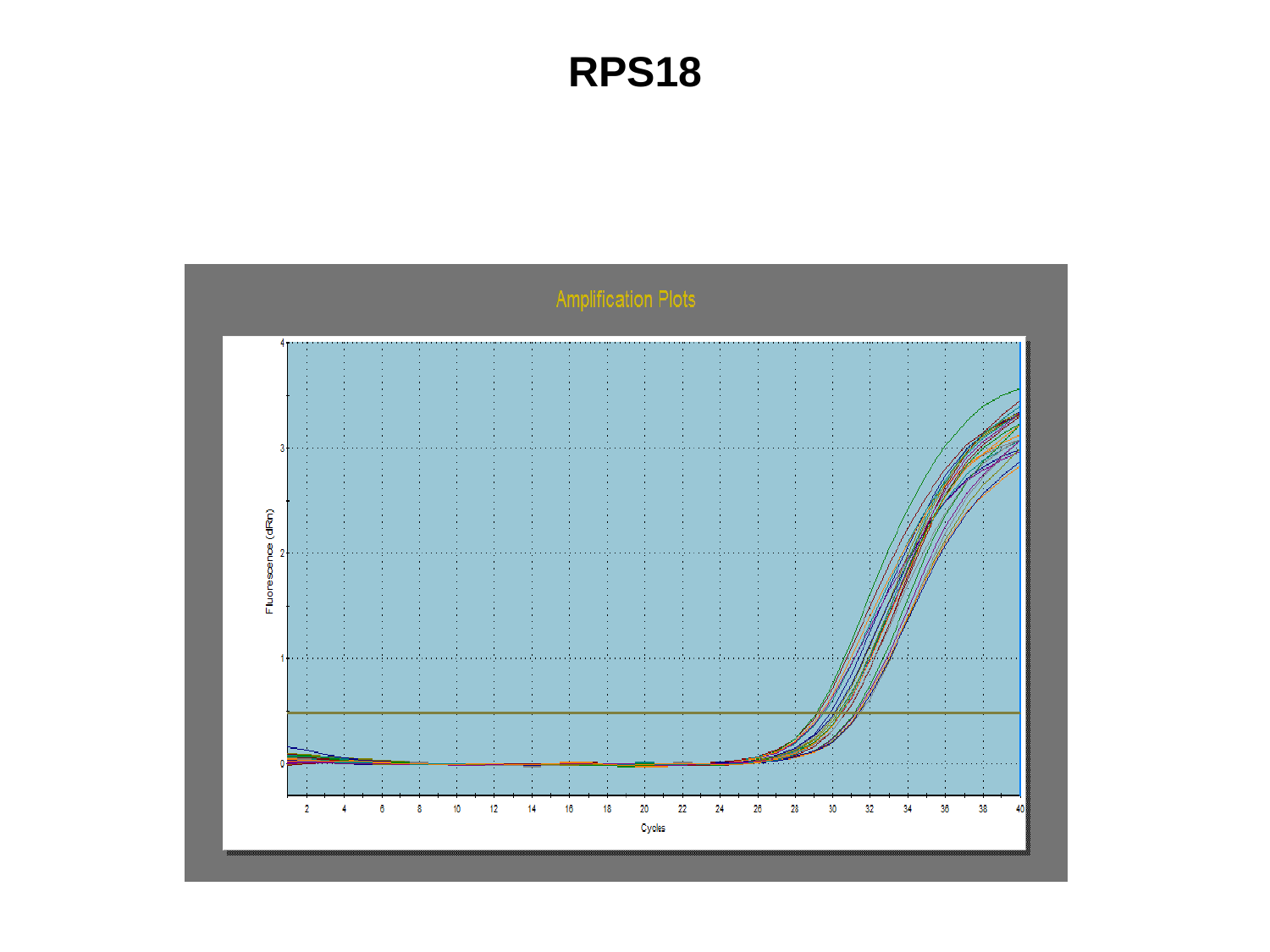

#
RPS18

## Slide 10
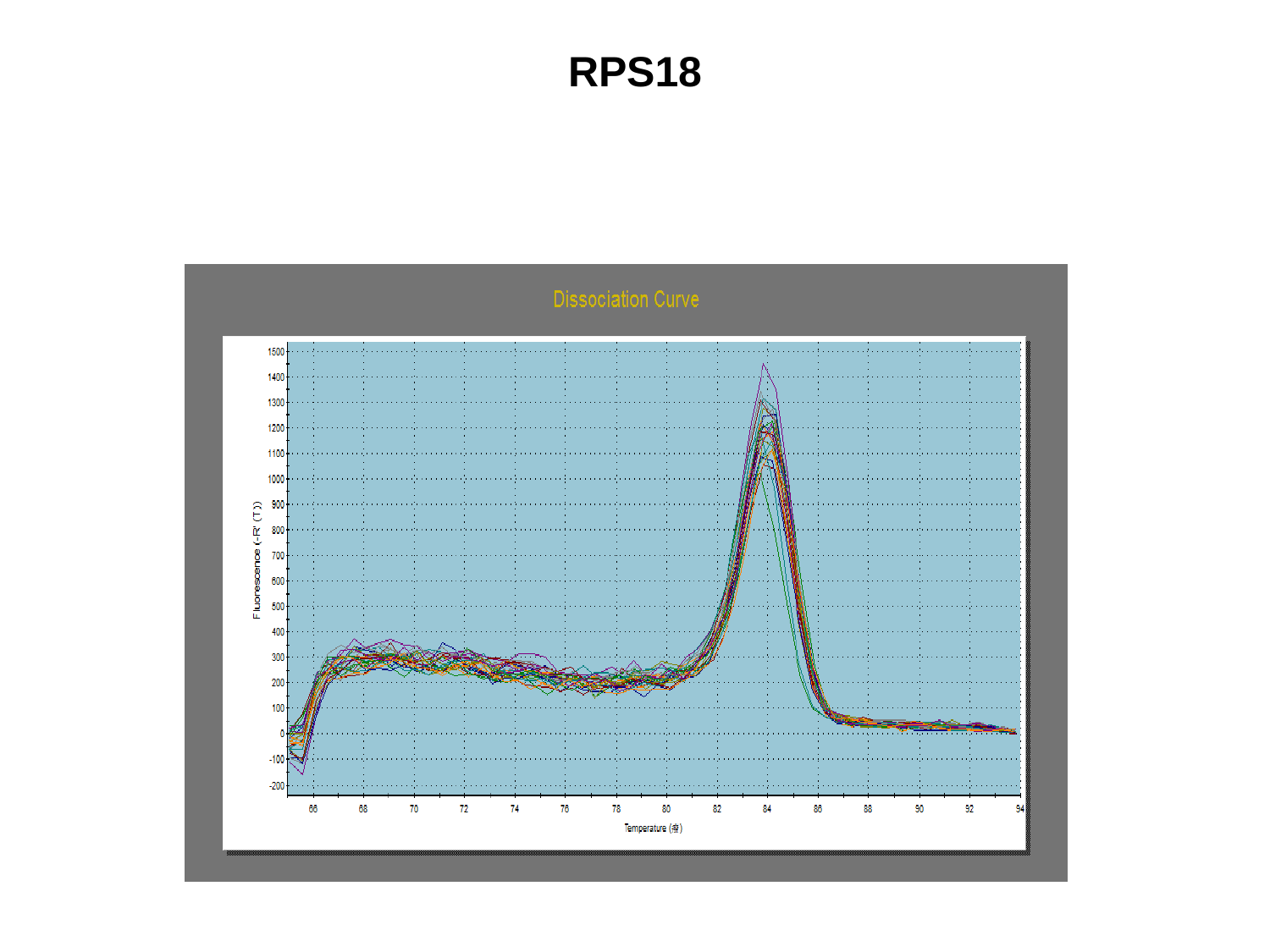

#
RPS18
